# The Autoimmune Manifestations in Patients with Genetic Defects in the B Cell Development and Differentiation Stages

**DOI:** 10.1007/s10875-023-01442-6

**Published:** 2023-02-15

**Authors:** Gholamreza Azizi, Mina Fattah Hesari, Niusha Sharifinejad, Farimah Fayyaz, Zahra Chavoshzadeh, Seyed Alireza Mahdaviani, Mahnaz Seifi Alan, Mahnaz Jamee, Marzieh Tavakol, Homa Sadri, Ehsan Shahrestanaki, Mohammad Nabavi, Sareh Sadat Ebrahimi, Afshin Shirkani, Ahmad Vosughi Motlagh, Samaneh Delavari, Seyed Erfan Rasouli, Marzie Esmaeili, Fereshte Salami, Reza Yazdani, Nima Rezaei, Hassan Abolhassani

**Affiliations:** 1 Non-Communicable Diseases Research Center, Alborz University of Medical Sciences, Karaj, Iran; 2 Research Center for Immunodeficiencies, Pediatrics Center of Excellence, Children’s Medical Center, Tehran University of Medical Sciences, Tehran, Iran; 3 Colorectal Research Center, Iran University of Medical Sciences, Tehran, Iran; 4 Network of Immunity in Infection, Malignancy and Autoimmunity (NIIMA), Universal Scientific Education and Research Network (USERN), Tehran, Iran; 5 Pediatric Infections Research Center, Mofid Children’s Hospital, Shahid Beheshti University of Medical Sciences, Tehran, Iran; 6 Pediatric Respiratory Diseases Research Center, National Research Institute of Tuberculosis and Lung Diseases, Shahid Beheshti University of Medical Sciences, Tehran, Iran; 7 Cardiovascular Research Center, Alborz University of Medical Sciences, Karaj, Iran; 8 Pediatric Nephrology Research Center, Research Institute for Children’s Health, Shahid Beheshti University of Medical Sciences, Tehran, Iran; 9 Department of Epidemiology, School of Public Health, Iran University of Medical Science, Tehran, Iran; 10 Department of Allergy and Clinical Immunology, Rasool E Akram Hospital, Iran University of Medical Sciences, Tehran, Iran; 11 Department of Immunology and Allergy, Kerman University of Medical Sciences, Kerman, Iran; 12 Allergy and Clinical Immunology Department, School of Medicine, Bushehr University of Medical Science, Moallem St, Bushehr, Iran; 13 Department of Pediatrics, North Khorasan University of Medical Sciences, Bojnurd, Iran; 14 Department of Neurology, Thomas Jefferson University, Philadelphia, PA USA; 15 Department of Immunology, School of Medicine, Tehran University of Medical Sciences, Tehran, Iran; 16 Division of Clinical Immunology, Department of Biosciences and Nutrition, Karolinska Institutet, Karolinska University Hospital, NEO, Blickagangen 16, 14157 Huddinge, Stockholm, Sweden

**Keywords:** Inborn errors of immunity, Primary immunodeficiency, B cell, Autoimmunity, Antibody deficiency, Class switch recombination

## Abstract

**Purpose:**

Primary B cell defects manifesting as predominantly antibody deficiencies result from variable inborn errors of the B cell lineage and their development, including impairments in early bone marrow development, class switch recombination (CSR), or terminal B cell differentiation. In this study, we aimed to investigate autoimmunity in monogenic patients with B cell development and differentiation defects.

**Methods:**

Patients with known genetic defects in the B cell development and differentiation were recruited from the Iranian inborn errors of immunity registry.

**Results:**

A total of 393 patients with a known genetic defect in the B cell development and differentiation (257 males; 65.4%) with a median age of 12 (6–20) years were enrolled in this study. After categorizing patients, 109 patients had intrinsic B cell defects. More than half of the patients had defects in one of the *ATM* (85 patients), *BTK* (76 patients), *LRBA* (34 patients), and *DOCK8* (33 patients) genes. Fifteen patients (3.8%) showed autoimmune complications as their first manifestation. During the course of the disease, autoimmunity was reported in 81 (20.6%) patients at a median age of 4 (2–7) years, among which 65 patients had mixed intrinsic and extrinsic and 16 had intrinsic B cell defects. The comparison between patients with the mentioned four main gene defects showed that the patient group with LRBA defect had a significantly higher frequency of autoimmunity compared to those with other gene defects. Based on the B cell defect stage, 13% of patients with early B cell defect, 17% of patients with CSR defect, and 40% of patients who had terminal B cell defect presented at least one type of autoimmunity.

**Conclusion:**

Our results demonstrated that gene mutations involved in human B cell terminal stage development mainly *LRBA* gene defect have the highest association with autoimmunity.

**Supplementary Information:**

The online version contains supplementary material available at 10.1007/s10875-023-01442-6.

## Introduction

Primary B cell defects manifesting as predominantly antibody deficiencies are the most common type of inborn errors of immunity (IEI) disorders [1–3]. Diverse intrinsic and extrinsic genetic variations result in variable developmental and/or functional defects of the B cell lineage, including defects in early B cell development, class switch recombination (CSR), or terminal B cell differentiation causing agammaglobulinemia, hyper-IgM (HIgM) syndrome, or hypogammaglobulinemia, respectively [4, 5]. Mutations in Bruton tyrosine kinase (*BTK*) are the most common intrinsic gene defects (85%) that cause early B cell defects, while mutations in CD40 ligand (*CD40L*, 70%) and transmembrane activator and CAML interactor (*TACI*, 10%) are the most prevalent causes of class switching defects and terminal B cell defects, respectively [6]. However, B cell defects can be observed in many other monogenic defects due to extrinsic adaptive and innate immunity defects [7, 8]. This dysregulation constitutes a heterogeneous group of disorders, with considerable variability in clinical and immunological phenotypes, encompassing antibody production impairment and recurrent infection, as well as autoimmunity [9, 10].

Several studies have indicated that patients with B cell defects have increased susceptibility to autoimmune complications [11–13]. These autoimmune manifestations may even be the first presentation prior to a severe infection or as the only presentation of the disease [14]. It was reported that one-fourth of patients with B cell defect present autoimmune complications. Autoimmunity is more prevalent in common variable immunodeficiency (CVID) (~ 30%) than in patients with agammaglobulinemia (~ 15%), selective immunoglobulin A deficiency (SIgAD) (~ 10%), and CSR defects (~ 5%) [15, 16]. The most common autoimmune disorders in B cell defects are autoimmune cytopenias, including immune thrombocytopenic purpura (ITP) and autoimmune hemolytic anemia (AIHA), due to the lack of self-tolerance. The other recurrent immune diseases in B cell defects include autoimmune thyroid diseases, type 1 diabetes (T1D), rheumatoid arthritis (RA), inflammatory bowel diseases (IBD), alopecia areata, vitiligo, and glomerulonephritis [11].

In the current study, we aimed to investigate the prevalence of autoimmunity in monogenic patients with both intrinsic and extrinsic B cell defects and determine whether the clinical and immunological features of patients in the three stages of B cell developmental defects in terms of early, class switch recombination or terminal B cell differentiation would affect the phenotype of autoimmunity.

## Material and Methods

### Patients

All available patients with known genetic defects in the B cell development and differentiation stages were included from the cases registered in the national IEI registry at Children’s Medical Center Hospital in Iran. The diagnosis of IEI was established based on the updated clinical diagnostic criteria recommended by the European Society for Immunodeficiencies (ESID) [17] and the Middle East and North Africa Diagnosis and Management Guidelines for IEI [18]. Genes involved in different stages of B lymphocyte differentiation, as well as intrinsic and mixed extrinsic and intrinsic genes, were categorized according to Amirifar et al. [19]. The classification of intrinsic and mixed extrinsic and intrinsic genes is presented in Table S1. The included patients had one pathogenic mutation in the genes involved in the B cell development based on the American College of Medical Genetics and Genomics (ACMG) criteria as described previously [, 7, 8]. Patients with more than one gene defect or missing data were excluded. The patients were divided into main groups based on the mutant gene involved in B cell development and differentiation; then, the groups were compared according to the presence of autoimmunity. The inclusion/exclusion diagram of patients is presented in Figure S1. Written informed consent was obtained from patients/parents. This study is approved by the Ethics Committee of the Alborz University of Medical Sciences (Ethics approval code: IR.ABZUMS.REC.1400.093).

### Study Design

The information was collected retrospectively by reviewing the medical records available in the registry or direct interviews with patients and/or their parents. The collected information was comprised of demographic data, medical history, physical examination, immunological assays, and molecular findings. The patient’s medical history included the first presentation, immune-related presentations, and autoimmune or poly-autoimmune (more than one autoimmune manifestation) diseases. Demographic information included age, gender, age of onset, age of diagnosis, delay in diagnosis, and current life status. Laboratory data consisted of white blood cell (WBC), hemoglobin, T and B cell subsets (assessed by flow cytometric analysis), and serum immunoglobulin levels (examined by nephelometry and enzyme-linked immunosorbent assay (ELISA)). The autoimmune diagnosis was confirmed with a combination of clinical manifestations and complementary paraclinical findings, including pathological biopsy results obtained directly or through endoscopy and/or colonoscopy, laboratory tests (direct Coombs test, anti-nuclear antibody profile (ANA), fluorescent anti-nuclear antibody (FANA), double-stranded DNA (anti-dsDNA), and other specific autoantibodies) and radiological studies according to international standards as described previously [15].

### Statistical Analysis

Qualitative data were described as frequency (percentages) and quantitative data as mean ± standard deviation (SD) or median (interquartile range, IQR), as appropriate. A chi-square test or Fisher’s exact test was used to compare three stages of B cell development. The assumption of normality of variables was tested using the Shapiro–Wilk test. Mann–Whitney U-tests for nonparametric data and *t*-tests for parametric data were used to compare numerical variables. Statistical analysis was performed using the SPSS software package, version 22 (SPSS Inc., Chicago, IL, USA). A *P* value < 0.05 was considered statistically significant.

## Results

### Molecular Findings and Group Classification

A total of 393 patients with a known genetic defect in the B cell development and differentiation stages (257 males; 65.4%) with a median (IQR) age of 12 (6–20) years were enrolled in this study. The median (IQR) age at onset, age at IEI diagnosis, and diagnostic delay were 1 (0.4–2), 4 (1–7), and 2 (0.3–5) years, respectively. Overall, 265 patients (69%) were born to consanguineous parents. The patients’ characteristics are summarized in Table 1.

**Table 1:** Demographic data in patients with primary B cell defects

Parameters	Total	With autoimmunity	Without autoimmunity	*P* value	Early B cell defects (*n* = 138)				
					Total	With autoimmunity	Without autoimmunity	*P* value	
Sex ratio, M/F (*n* = 393)	257/136	46/35	211/101	0.068	111/27	13/5	98/22	0.346	
Age (y), median (IQR) (*n* = 387)	12 (6–20)	13.5 (8.3–20.8)	11 (6–20)	0.038*	10 (5–22)	14 (6–28.5)	10 (4.3–22)	0.203	
Age at onset (y), median (IQR) (*n* = 384)	1 (0.4–2)	1 (0.5–3)	1 (0.3–2)	0.023*	0.6 (0.2–1.3)	1 (0.5–1.6)	0.5 (0.2–1.3)	0.232	
Age at diagnosis of IEI (y), median (IQR) (*n* = 376)	4 (1–7)	6 (3–8.8)	4 (1–7)	0.003*	3 (1–6)	5 (1–7)	2 (1–6)	0.201	
Delay in diagnosis (y), median (IQR) (*n* = 374)	2 (0.3–5)	3 (0.4–5.3)	2 (0.3–4.5)	0.195	1.1 (0.2–4)	2.7 (0.2–5.2)	1 (0.2–4)	0.569	
Course of disease (y), median (IQR) (*n* = 382)	9 (3.5–16)	10 (5.3–17.8)	8.4 (3–15)	0.060	7.7 (1.1–18.3)	10.1 (3.5–27.1)	6.6 (1–17.8)	0.230	
Consanguinity (%) (*n* = 384)	265 (69%)	56 (21.1%)	209 (78.9%)	0.868	69 (51.1%)	10 (14.5%)	59 (85.5%)	0.496	
Dead/alive ratio** (*n* = 381)	83/297	17/58	66/239	0.686	30/105	1/16	29/89	0.205	

Parameters	CSR defects (*n* = 140)				Terminal B cell defects (*n* = 115)				*P* value
	Total	With autoimmunity	Without autoimmunity	*P* value	Total	With autoimmunity	Without autoimmunity	*P* value	
Sex ratio, M/F (*n* = 393)	81/59	11/13	70/46	0.190	65/50	22/17	43/33	0.986	<0.001*
Age (y), median (IQR) (*n* = 387)	12 (8–16)	11 (9–17.8)	12 (8–16)	0.916	12 (6–22)	15 (9–22)	12 (6–21.8)	0.166	0.235
Age at onset (y), median (IQR) (*n* = 384)	1 (0.5–2)	1.3 (0.5–3.4)	1 (0.5–2)	0.343	1 (0.5–3)	2 (0.5–5)	1 (0.5–3)	0.410	<0.001*
Age at diagnosis of IEI (y), median (IQR) (*n* = 376)	4 (2–7)	5 (3–7.0)	4 (2–7)	0.423	6 (2.3–11	7.5 (4–13)	5.9 (1.9–11)	0.118	0.534
Delay in diagnosis (y), median (IQR) (*n* = 374)	2 (0.5–4.1)	2.5 (0.6–4)	2 (0.5–4.4)	0.864	3.4 (0.5–6)	3.8 (0.4–6.1)	2.9 (0.4–6.5)	0.567	0.023*
Course of disease (y), median (IQR) (*n* = 382)	9 (5.5–13.5)	8.5 (5.3–11)	9 (5.4–14)	0.799	9.5 (3–18)	12.4 (5.4–19)	8.3 (3–17.7)	0.129	0.134
Consanguinity (%) (*n* = 384)	108 (78.3%)	17 (15.7%)	91 (84.3%)	0.332	88 (79.3)	29 (33%)	59 (67%)	0.578	<0.001*
Dead/alive ratio** (*n* = 381)	32/104	5/17	27/87	0.923	21/88	11/25	10/63	0.036*	0.650

*F* female, *M* male, *Y* year, *IEI* inborn errors of immunity, *IQR *Interquartile range, *CSR *class-switch recombination

^*^Bold values indicate *P* < 0.05 and are considered significant

^**^Alive/dead data are not available for 12 patients

Our genetics analysis identified 37 mutated genes with different inheritance patterns and various types of mutations (Table S2). Regarding the role of the identified gene in B cell development [19], 21 genes were involved in the terminal stage (115 patients), nine genes in the early stage (138 patients), and seven genes in the CSR stage (140 patients). Furthermore, the most frequent mutated genes associated with each stage of B cell development were as follows: early: *BTK* (55.1%), *RAG1* (9.4%), and *IGHM* (10.1%); CSR: *ATM* (60.7%), *CD40L* (18.6%), and *DNMT3B* (7.1%); terminal stage: *LRBA* (29.6%), *DOCK8* (28.7%), and *WAS* (10.4%) (Table 2). When categorized based on the intrinsic and extrinsic B cell gene defects, 284 patients had mixed intrinsic and extrinsic gene defects, whereas 109 patients had intrinsic gene defects (Tables S3–9).

**Table 2:** Frequency of autoimmune disease in patients with B cell defects, overall and by the mutated gene

Mutated gene	*N*	Autoimmunity	
		Overall	Poly
Early stage			
All	138	18 (13%)	5/18
*BTK*	76	9 (11.8%)	2/9
*IGHM*	14	4 (28.6%)	1/4
*RAG1*	13	2 (15.4%)	1/2
*ADA*	11	-	-
*RAG2*	10	-	-
*DCLRE1C*	8	1 (12.5%)	1/1
*BLNK*	2	1 (50%)	-
*NHEJ1*	2	1 (50%)	-
*CD79A*	2	-	-
CSR stage			
All	140	24 (17%)	5/24
*ATM*	85	13 (15.3%)	3/13
*CD40L*	26	5 (19.2%)	1/5
*DNMT3B*	10	2 (20%)	1/2
*AICDA*	10	1 (10%)	-
*ZBTB24*	6	2 (33.3%)	-
*IKBKB*	2	1 (50%)	-
*IKBKG*	1	-	-
Terminal stage			
All	115	39 (40%)	16/39
*CD70*	1	1 (100%)	1/1
*NFKB1*	1	1 (100%)	-
*SH2DA1*	1	1 (100%)	-
*LRBA*	34	24 (70.6%)	13/24
*WAS*	12	3 (25%)	-
*CD27*	4	1 (25%)	-
*XIAP*	3	1 (33.3%)	1/1
*CTLA4*	3	1 (33.3%)	1/1
*DOCK8*	33	1 (3%)	-
*PIK3CD*	2	1 (50%)	-
*RAC2*	3	-	-
*NFKB2*	2	-	-
*CARD11*	1	-	-
*ICOS*	1	-	-
*TPP2*	1	-	-
*TACI*	1	-	-
*TTC7A*	1	-	-
*PRKCD*	1	-	-
*BAFFR*	3	1 (33.3%)	-
*PIK3R1*	3	1 (33.3%)	-
*STAT1*	3	2 (66.7%)	-

*CSR* class switch recombination, *N* number, *Poly* poly-autoimmunity, CSR class-switch recombination

### Clinical Presentation History

**Fig. 1:**
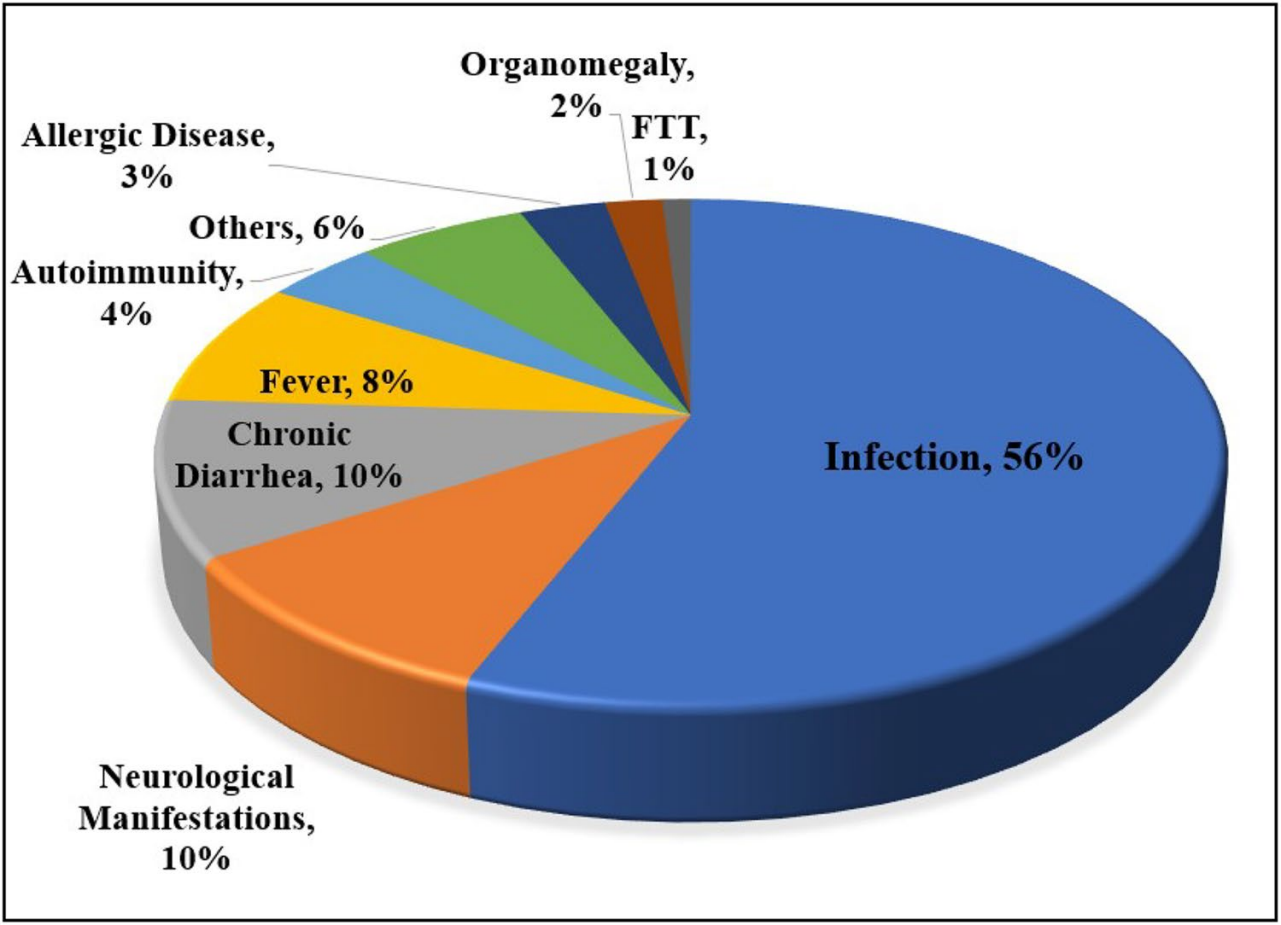
First presentation in 393 patients with primary B cell defects. FTT failure to thrive. Others included presentations such as convulsion, arthritis, faintness, facial nerve palsy, skin lesion, imbalance, stagger, coughing, inguinal hernia, gastrointestinal bleeding, jaundice and anorexia

As shown in Fig. 1, the most prevalent first presentations in this cohort were infectious diseases (*n* = 215, 56%), followed by neurological manifestations and chronic diarrhea (each in 37 patients, 9.4%). Fifteen patients (3.8%) showed autoimmune complications (mostly ITP) as their first presentation. Infection was also the most prevalent first manifestation in each stage of B cell development.

The clinical manifestations of patients with B cell defects are presented in Table 3. Autoimmunity was reported in 81 (20.6%, 46 of which were male) patients at a median (IQR) age of 4 (2–7) years. Of the 81 patients with autoimmunity, 26 (32.1%) developed poly-autoimmunity (more than one type of autoimmunity). The first episode of autoimmunity in 55.1% of IEI patients with autoimmunity was diagnosed before the IEI, and in 10.3% of patients, IEI and autoimmunity were diagnosed at the same time. The median (IQR) age at onset in patients with autoimmunity was 2 (0.5–3) years, while in patients without autoimmunity was 1 (0.3–2) years (*P* = 0.023). The diagnosis of B cell defect was at a later age in patients with any autoimmunity presentations in the course of their lives (6 (3–8.8) vs. 4 (1–7) years, *P* = 0.003) with a slightly higher diagnostic delay compared to patients without autoimmunity. Age at onset, age at IEI diagnosis, and diagnostic delay of B cell development stages were not significantly different in patients with and without autoimmunity.

**Table 3:** Clinical manifestations in patients with primary B cell defects

Parameters	Total	With autoimmunity	Without autoimmunity	*P* value	Early B cell defects				
					Total	With autoimmunity	Without autoimmunity	*P* value	
Infectious manifestation, *n* (%)	323 (83.5%)	70 (21.7%)	253 (78.3%)	0.314	116 (84.7%)	15 (12.9%)	101 (87.1%)	1.000	
Otitis media, *n* (%)	149 (38.6%)	37 (24.8%)	112 (75.2%)	0.123	50 (36.5%)	8 (16%)	42 (84%)	0.446	
Sinusitis, *n* (%)	114 (29.5%)	35 (30.7%)	79 (69.3%)	0.002*	44 (31.9%)	10 (22.7%)	34 (77.3%)	0.030*	
Pneumonia, *n* (%)	211 (54.4%)	47 (22.3%)	164 (77.7%)	0.450	83 (60.1)	11 (13.3%)	72 (86.7%)	1.000	
Skin infection, *n* (%)	66 (17%)	12 (18.2%)	54 (81.8%)	0.738	16 (11.6%)	3 (18.8%)	13 (81.3%)	0.440	
Candidiasis, *n* (%)	45 (11.6%)	14 (31.1%)	31 (68.9%)	0.078	15 (10.9%)	2 (13.3%)	13 (86.7%)	1.000	
Conjunctivitis, *n* (%)	44 (11.4%)	11 (25%)	33 (75%)	0.434	19 (13.8%)	3 (15.8%)	16 (84.2%)	0.715	
Meningitis, *n* (%)	35 (9%)	9 (25.7%)	26 (74.3%)	0.511	18 (13%)	2 (11.1%)	16 (88.9%)	1.000	
Septicemia, *n* (%)	7 (1.8%)	4 (57.1%)	3 (42.9%)	0.036*	1 (0.7%)	0 (0.0%)	1 (100%)	1.000	
Septic arthritis, *n* (%)	18 (4.6%)	8 (44.4%)	10 (55.6%)	0.017	3 (2.2%)	1 (33.3%)	2 (66.7%)	0.345	
Bronchiectasis, *n* (%)	64 (16.5%)	20 (31.3%)	44 (68.8%)	0.028*	27 (19.6%)	5 (18.5%)	22 (81.5%)	0.348	
Neutropenia, *n* (%)	36 (9.4%)	7 (19.4%)	29 (80.6%)	1.000	12 (8.7%)	1 (8.3%)	11 (91.7%)	1.000	
Failure to thrive, *n* (%)	74 (19.1%)	17 (23%)	57 (77%)	0.632	18 (13%)	1 (5.6%)	17 (94.4%)	0.467	
Lymphoproliferation, *n* (%)	108 (27.9%)	46 (42.6%)	62 (57.4%)	< 0.001*	29 (21.0%)	6 (20.7%)	23 (79.3%)	0.169	
Splenomegaly, *n* (%)	71 (18.3%)	37 (52.1%)	34 (47.9%)	< 0.001*	16 (11.6%)	3 (18.8%)	13 (81.2%)	0.440	
Hepatomegaly, *n* (%)	59 (15.2%)	25 (42.4%)	34 (57.6%)	< 0.001*	19 (13.8%)	4 (21.1%)	15 (78.9%)	0.275	
Lymphadenopathy, *n* (%)	57 (14.7%)	23 (40.4%)	34 (59.6%)	< 0.001*	17 (12.3%)	2 (11.8%)	15 (88.2%)	1.000	
Clubbing, *n* (%)	35 (9%)	14 (40%)	21 (60%)	0.007*	11 (8%)	2 (18.2%)	9 (81.8%)	0.637	
Malignancy, *n* (%)	11 (2.8%)	4 (36.44%)	7 (63.6%)	0.249	1 (0.7%)	1 (100%)	0	0.130	
Enteropathy,* n* (%)	87 (22.5%5)	34 (39.1%)	53 (60.9%)	< 0.001*	40 (29%)	8 (20%)	32 (80%)	0.162	
Allergy/asthma, *n* (%)	51 (13.2%)	14 (27.5%)	37 (72.5%)	0.198	12 (8.7%)	3 (25%)	9 (75%)	0.192	

Parameters	CSR defects				Terminal B cell defects				*P* value
	Total	With autoimmunity	Without autoimmunity	*P* value	Total	With autoimmunity	Without autoimmunity	*P* value	
Infectious manifestation, *n* (%)	109 (79%)	21 (19.3%)	88 (80.7%)	0.408	98 (87.5%)	34 (34.7%)	64 (65.3%)	0.769	0.176
Otitis media, *n* (%)	58 (42%)	13 (22.4%)	45 (77.6%)	0.255	41 (36.9%)	15 (39%)	25 (61%)	0.534	0.586
Sinusitis, *n* (%)	28 (20.3%)	8 (28.6%)	20 (71.4%)	0.096	42 (37.8%)	17 (40.5%)	25 (59.5%)	0.308	0.008*
Pneumonia, *n* (%)	68 (49.3%)	13 (19.1%)	55 (80.9%)	0.657	60 (53.6)	23 (38.3%)	37 (61.7%)	0.322	0.189
Skin infection, *n* (%)	19 (13.8%)	4 (21.1%)	15 (78.9%)	0.744	31 (27.7%)	5 (16.1%)	26 (83.9%)	0.015	0.002*
Candidiasis, *n* (%)	5 (3.6%)	2 (40%)	3 (60%)	0.208	25 (22.5%)	10 (40%)	15 (60%)	0.485	< 0.001*
Conjunctivitis, *n* (%)	13 (9.4%)	3 (23.1%)	10 (76.9%)	0.699	12 (10.8%)	5 (41.7%)	7 (58.3%)	0.541	0.511
Meningitis, *n* (%)	3 (2.2%)	0 (0%)	3 (100%)	1.000	14 (12.6%)	7 (50%)	7 (50%)	0.231	0.002*
Septicemia, *n* (%)	3 (2.2%)	2 (66.7%)	1 (33.3%)	0.078	3 (2.7%)	2 (66.7%)	1 (33.3%)	0.269	0.469
Septic arthritis, *n* (%)	4 (2.9%)	1 (25%)	3 (75%)	0.539	11 (9.8%)	6 (54.5%)	5 (45.5%)	0.179	0.008*
Bronchiectasis, *n* (%)	11 (8%)	2 (18.2%)	9 (81.8%)	1.000	26 (23.4%)	13 (50%)	13 (50%)	0.062	0.002*
Neutropenia, *n* (%)	22 (15.9%)	6 (27.3%)	16 (72.7%)	0.219	2 (1.9%)	0 (0.0%)	2 (100.0%)	1.000	0.001*
Failure to thrive, *n* (%)	29 (21%)	4 (13.8%)	25 (86.2%)	0.784	27 (24.3%)	12 (44.4%)	15 (55.6%)	0.245	0.062
Lymphoproliferation, *n* (%)	33 (23.9%)	13 (39.4%)	20 (60.6%)	< 0.001*	46 (41.4%)	13 (39.4%)	20 (60.6%)	< 0.001*	0.001*
Splenomegaly, *n* (%)	22 (15.9%)	11 (50%)	11 (50%)	< 0.001*	33 (29.7%)	23 (69.7%)	10 (30.3%)	< 0.001*	0.001*
Hepatomegaly, *n* (%)	13 (9.4%)	7 (53.8%)	6 (46.2%)	0.002*	27 (24.3%)	14 (51.9%)	13 (48.1%)	0.036*	0.004*
Lymphadenopathy, *n* (%)	21 (15.2%)	6 (28.6%)	15 (71.4%)	0.206	19 (17.1%)	15 (78.9%1	4 (21.1%)	< 0.001*	0.558
Clubbing, *n* (%)	6 (4.3%)	1 (16.7%)	5 (83.3%)	1.000	18 (16.2%)	11 (61.1%)	7 (38.9%)	0.014*	0.004*
Malignancy, *n* (%)	6 (4.3%)	1 (16.7%)	5 (83.3%)	1.000	4 (3.6%)	2 (50.0%)	2 (50.0%)	0.605	0.165
Enteropathy,* n* (%)	12 (8.7%)	2 (16.7%)	10 (83.3%)	1.000	35 (31.5%)	24 (68.6%)	11 (31.4%)	< 0.001*	< 0.001*
Allergy/asthma, *n* (%)	11 (8%)	2 (18.2%)	9 (81.8%)	1.000	28 (25.2%)	9 (32.1%)	19 (67.9%)	0.823	< 0.001*

* and bold values indicate *P* < 0 .05 and are considered significant, CSR class-switch recombination

Autoimmune diseases involved hematologic (46.9%), rheumatologic (28.4%), gastrointestinal (21%), dermatologic (16%), neurologic (7.4%), and endocrine (6.2%) systems. The most commonly reported types of autoimmune disorders were ITP (7.9%), juvenile idiopathic arthritis (JIA) (5.3%), and AIHA (4.8%) (Table 4). Among patients with autoimmunity, 65 patients (80.2%) had mixed intrinsic and extrinsic and 16 patients (19.8%) had B cell intrinsic gene defects. ITP and AIHA frequencies were significantly higher in patients with mixed intrinsic and extrinsic gene defects compared to those with intrinsic gene defects (Table 5), and in general, the highest number of patients in most autoimmune diseases was from the terminal B cell developmental stage defects. Among patients with the main gene defects, those with *LRBA* mutation had the highest frequency of autoimmunity, accounting for 70.6% of patients who had autoimmunity presentations; furthermore, over half of the *LRBA* deficient patients had more than one autoimmune disease (Tables 2 and S7). Among patients with autoimmune diseases, 15.4% of patients with B cell defects at an early stage (8 out of 18), 30.8% at the CSR stage (16 out of 24), and 53.9% at the terminal stage (28 out of 39) had autoimmunities with specific diagnostic autoantibodies (celiac disease, systemic lupus erythematosus, autoimmune hepatitis, and RA). The frequency of autoimmunities with specific autoantibodies was not significantly different between the groups (*P* = 0.129).

**Table 4:** Autoimmune manifestation in patients with primary B cell defects

Parameters	Total	Mono-autoimmunity	Poly-autoimmunity	*P* value	Early B cell defects				
					Total	Mono-autoimmunity	Poly-autoimmunity	*P* value	
Immune thrombocytopenic purpura	31 (7.9%)	14 (45.2%)	17 (54.8%)	< 0.001*	3 (9.7%)	0	3	0.000*	
Autoimmune hemolytic anemia	19 (4.8%)	5 (26.3%)	14 (73.7%)	< 0.001*	2 (10.5%)	1	1	0.071	
Autoimmune enteropathy	5 (1.3%)	1 (20.0%)	4 (80.0%)	< 0.001*	0 (0.0%)	0	0	–––-	
Rheumatoid arthritis/juvenile idiopathic arthritis	21 (5.3%)	13 (61.9%)	8 (38.1%)	< 0.001*	8 (38.1%)	5	3	0.001*	
Autoimmune thyroiditis	3 (0.8%)	0 (0.0%)	3 (100%)	< 0.001*	0 (0.0%)	0	0	–––-	
Vitiligo	6 (1.5%)	3 (50%)	3 (50%)	0.005*	1 (16.7%)	1	0	1.000	
Insulin-dependent diabetes mellitus	2 (0.5%)	2 (100%)	0 (0.0%)	1.000	0 (0.0%)	0	0	–––-	
Autoimmune Addison disease	0 (0.0%)	0 (0.0%)	0 (0.0%)	–––	0	0	0	–––-	
Celiac disease	4 (1%)	2 (50%)	2 (50%)	0.023*	1 (25%)	0	1	0.036*	
Guillain-Barré syndrome	4 (1%)	4 (100%)	0 (0.0%)	1.000	2 (50%)	2	0	1.000	
Alopecia areata	4 (1%)	1 (25%)	3 (75%)	0.001*	1 (25%)	1	0	1.000	
Inflammatory bowel disease	8 (2%)	3 (37.5%)	5 (62.5%)	< 0.001*	0 (0.0%)	0	0	–––-	
Myasthenia gravis	1 (0.3%)	0 (0.0%)	1 (100%)	0.066	0 (0.0%)	0	0	–––-	
Systemic lupus erythematous	3 (0.8%)	1 (33.3%)	2 (66.7%)	0.012*	1 (33.3%)	1	0	1.000	
Psoriasis	4 (1%)	3 (75%)	1 (25%)	0.240	2 (50%)	1	1	0.071	
Kawasaki disease	2 (0.5%)	1 (50%)	1 (50%)	0.124	2 (100%)	1	1	0.071	
Evans syndrome	2 (0.5)	0 (0.0%)	2 (100%)	0.004*	0 (0.0%)	0	0	–––-	
Multiple sclerosis	1 (0.3%)	0 (0.0%)	1 (100%)	0.066	0 (0.0%)	0	0	–––-	
Autoimmune hepatitis	3 (0.8%)	2 (66.7%)	1 (33.3%)	0.186	0 (0.0%)	0	0	–––-	

Parameters	CSR defects				Terminal B cell defects				*P* value
	Total	Mono-autoimmunity	Poly-autoimmunity	*P* value	Total	Mono-autoimmunity	Poly-autoimmunity	*P* value	
Immune thrombocytopenic purpura	9 (29.0%)	7	2	0.033*	19 (61.3%)	7	12	< 0.001*	< 0.001*
Autoimmune hemolytic anemia	5 (26.3%)	3	2	0.010*	12 (63.2%)	1	11	< 0.001*	0.003*
Autoimmune enteropathy	0 (0.0%)	0	0	–––-	5 (100%)	1	4	0.001*	0.002*
Rheumatoid arthritis/juvenile idiopathic arthritis	7 (33.3%)	3	4	< 0.001*	6 (28.6%)	5	1	1.000	0.955
Autoimmune thyroiditis	1 (33.3%)	0	1	0.036*	2 (66.7%)	0	2	0.018*	0.286
Vitiligo	1 (16.7%)	0	1	0.036*	4 (66.7%)	2	2	0.092	0.128
Insulin-dependent diabetes mellitus	0 (0.0%)	0	0	–––-	2 (100%)	2	0	1.000	0.088
Autoimmune Addison disease	0	0	0	–––-	0	0	0	–––-	–––
Celiac disease	0 (0.0%)	0	0	–––-	3 (75%)	2	1	0.365	0.108
Guillain-Barré syndrome	2 (50%)	2	0	1.000	0 (0.0%)	0	0	–––-	0.433
Alopecia areata	0 (0.0%)	0	0	––––	3 (75%)	0	3	0.002*	0.108
Inflammatory bowel disease	4 (50%)	2	2	0.006*	4 (50%)	1	3	0.008*	0.103
Myasthenia gravis	0 (0.0%)	0	0	–––-	1 (100%)	0	1	0.139	0.298
Systemic lupus erythematous	2 (66.7%)	0	2	0.001*	0 (0.0%)	0	0	–––-	0.426
Psoriasis	1 (25%)	1	0	1.000	1 (25%)	1	0	1.000	0.815
Kawasaki disease	0 (0.0%)	0	0	–––-	0 (0.0%)	0	0	–––-	0.158
Evans syndrome	0 (0.0%)	0	0	–––-	2 (100%)	0	2	0.018*	0.088
Multiple sclerosis	0 (0.0%)	0	0	–––-	1 (100%)	0	1	0.139	0.298
Autoimmune hepatitis	1 (33.3%)	1	0	1.000	2 (66.7%)	1	1	0.260	0.285

^*^ and bold values indicate *P* < 0.05 and are considered significant, CSR class-switch recombination

**Table 5:** Autoimmunity in intrinsic and mixed gene groups

Parameters	Mixed intrinsic and extrinsic	Intrinsic	*P* value
Autoimmunity (*n* = 81)	65 (80.2%)	16 (19.8%)	0.094
Immune thrombocytopenic purpura (*n* = 31)	30 (96.8%)	1 (3.2%)	0.001*
Autoimmune hemolytic anemia (*n* = 19)	19 (100%)	0	0.003*
Autoimmune enteropathy (*n* = 5)	5 (100%)	0	0.328
Rheumatoid arthritis/juvenile idiopathic arthritis (*n* = 21)	12 (57.1%)	9 (42.9%)	0.133
Autoimmune thyroiditis (*n* = 3)	3 (100%)	0	0.563
Vitiligo (*n* = 6)	5 (83.3%)	1 (16.7%)	1.000
Insulin-dependent diabetes mellitus (*n* = 2)	2 (100%)	0	1.000
Celiac disease (*n* = 4)	4 (100%)	0	0.579
Guillain-Barré syndrome (*n* = 4)	2 (50%)	2 (50%)	0.308
Alopecia areata (*n* = 4)	3 (75%)	1 (25%)	1.000
Inflammatory bowel disease (*n* = 8)	8 (100%)	0	0.113
Myasthenia gravis (*n* = 1)	1 (100%)	0	1.000
Systemic lupus erythematous (*n* = 3)	2 (66.7%)	1 (33.3%)	1.000
Psoriasis (*n* = 4)	2 (50%)	2 (50%)	0.308
Kawasaki disease (*n* = 2)	0	2 (100%)	0.077
Evans syndrome (*n* = 2)	2 (100%)	0	1.000
Multiple sclerosis (*n* = 1)	1 (100%)	0	1.000
Autoimmune hepatitis (*n* = 3)	3 (100%)	0	0.564

Evidence of infection was reported in 83.5% (*n* = 323) of the patients and 21.7% (*n* = 70) of them had concomitant autoimmunity. The most common infections were pneumonia (54.4%), otitis media (38.6%), and sinusitis (29.5%), and only 1.8% of patients had sepsis. There was no significant difference between the infection frequencies in each of the three B cell development stage groups. In each stage, pneumonia was the most prevalent infection. Non-neoplastic lymphoproliferation, including lymphadenopathy, splenomegaly, and hepatomegaly, was observed in 108 patients (27.9%), and its rate was significantly higher in patients with autoimmunity (*P* < 0.001). Moreover, the frequency rate of lymphoproliferation was significantly higher in the terminal stage (42.4%) in comparison with other groups (*P* = 0.001). Among other non-infectious manifestations, clubbing and enteropathy were significantly more likely present in patients with autoimmunity and terminal stage B cell defects. In contrast, there was no significant difference in the malignancy rate between patients with and without autoimmunity or at different B cell defect stages.

**Fig. 2:**
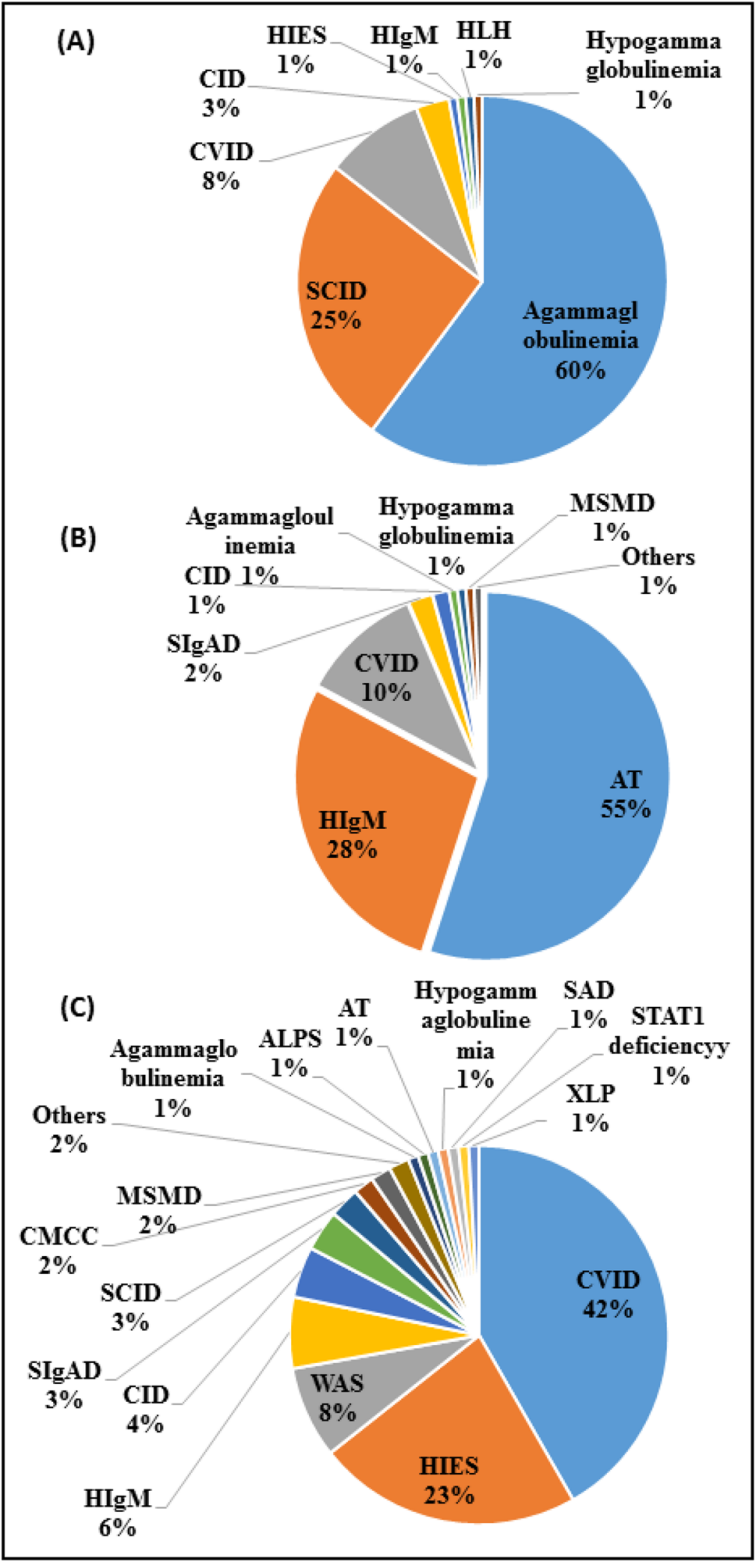
The spectrum of clinical diagnoses in B cell defective patients in early stage (**A**, *n* = 138), CSR stage (**B**, *n* = 140), and terminal stage (**C**, *n* = 115) of B cell developmental defect. CVID common variable immunodeficiency, HIgM hyper-IgM syndrome, HIES hyper-IgE syndrome, MSMDs Mendelian susceptibility to mycobacterial diseases, SIgAD selective IgA deficiency, WAS Wiskott-Aldrich syndrome, AT ataxia-telangiectasia, SCID severe combined immunodeficiency, SAD specific antibody deficiency, CID combined immunodeficiency, CMCC chronic mucocutaneous candidiasis, XLP X-linked lymphoproliferative, ALPS autoimmune lymphoproliferative syndrome, HLH hemophagocytic lymphohistiocytosis

**Fig. 3:**
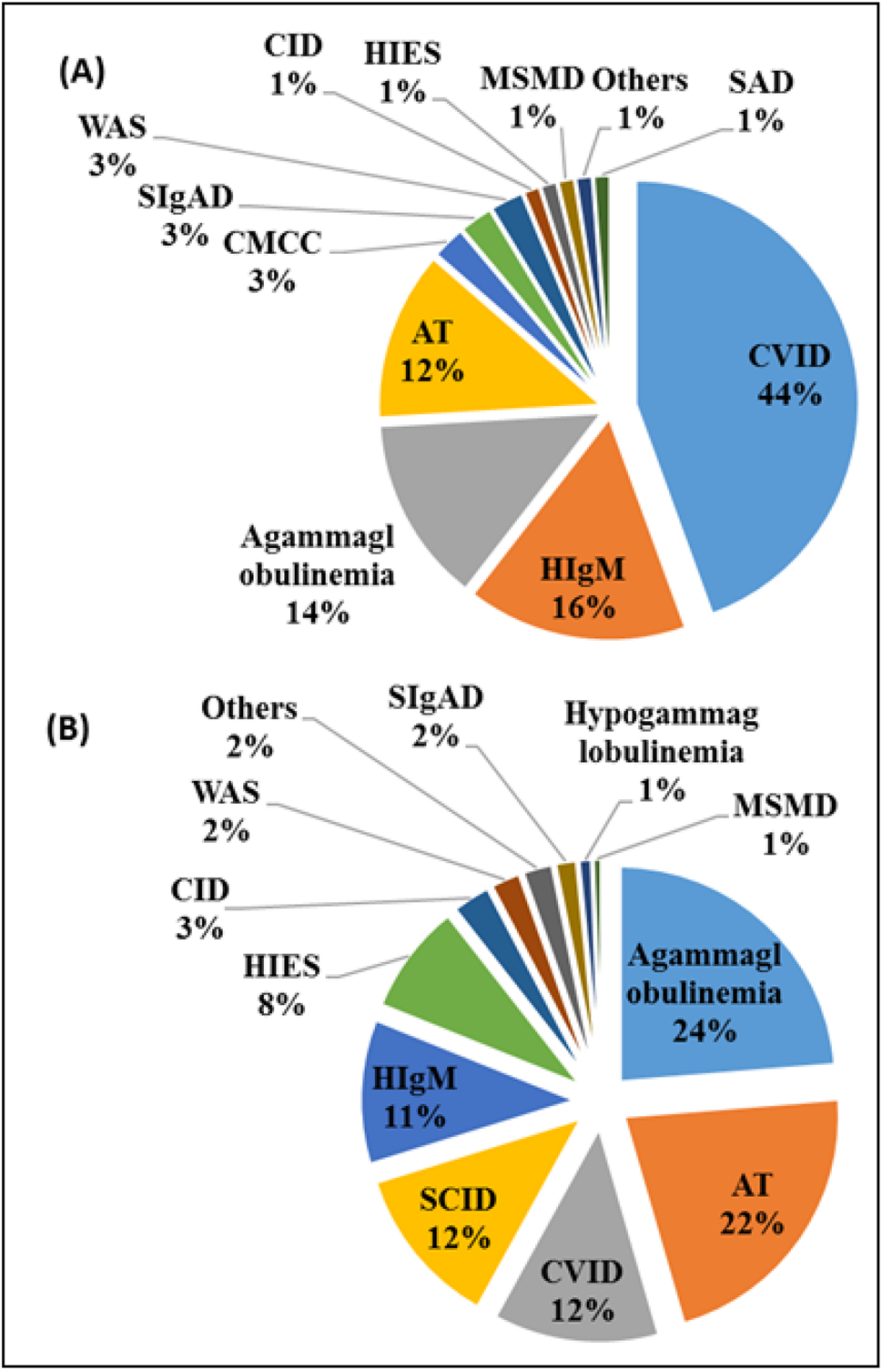
The spectrum of clinical diagnoses in B cell defective patients with autoimmunity (***A***, *n* = 81) and without autoimmunity (***B***, *n* = 312). CVID common variable immunodeficiency, HIgM hyper-IgM syndrome, HIES hyper-IgE syndrome, MSMDs Mendelian susceptibility to mycobacterial diseases, SIgAD selective IgA deficiency, WAS Wiskott-Aldrich syndrome, AT ataxia-telangiectasia, SCID severe combined immunodeficiency, SAD specific antibody deficiency, CID combined immunodeficiency, CMCC chronic mucocutaneous candidiasis

The most prevalent clinical diagnoses in the early stage were agammaglobulinemia (58%) and severe combined immunodeficiency (SCID) (25.4%), while patients with CSR defects were mainly diagnosed with ataxia-telangiectasia (AT, 55%) and HIgM (27.9%). In the terminal stage, CVID (41.7%) and hyper-IgE syndrome (HIES) were the most common clinical diagnoses (Fig. 2). Most of the patients with B cell defects and autoimmunity were initially diagnosed with the clinical impression of CVID (44.4%), HIgM syndrome (16.1%), and agammaglobulinemia (13.6%). In patients without autoimmunity, the main first clinical diagnoses were agammaglobulinemia (22.4%), AT (21.8%), and CVID (12.5%) (Fig. 3).

### Immunologic Evaluation

The immunological findings of patients with IEI are summarized in Table 6. Lymphocyte count was within the normal range in 42% (144 of 343 with available data) of patients, and lymphopenia was reported in 18.1% (62 of 343). Most of the patients had normal lymphocyte subsets, including CD3 + (53.6% (179 of 334)), CD4 + (59.4% (196 of 330)), CD8 + (52% (168 of 323)), CD19 + (37.2% (121 of 325)), and CD16 + 56 + (60.8% (101 of 166)). IEI patients with autoimmunity had a lower frequency of CD16 + 56 + NK cells than patients without autoimmunity (*P* = 0.016), while the number of CD19 + B cells was higher in IEI patients with autoimmunity than without (*P* = 0.040). The majority of patients had low levels of serum IgG (59.2% (174 of 325)), IgA (63.4% (232 of 366)), and IgM (40.8% (149 of 365)). About 23% of patients had a high serum level of IgM (84 of 365 patients with available data), while 36.2% had a normal serum level of IgM. The prevalence of patients with low IgG, IgA, and IgM serum levels was higher in the autoimmunity group than in the without group.

**Table 6:** Immunologic profile in patients with primary B cell defects

Parameters; median, (IQR)	Total	With autoimmunity	Without autoimmunity	*P* value	Early B cell defects (*n* = 138)				
					Total	With autoimmunity	Without autoimmunity	*P* value	
WBC × 1000 (cell/μL)	8 (5.5–11.5)	6.95 (4.94–11.12)	8.30 (5.66–11.60)	0.063	8.4 (5.8–13)	10.9 (4.1–14.6)	8.2 (5.8–11.8)	0.564	
Hemoglobin (g/dL)	11.3 (10–13)	11.15 (10–13)	11.60 (10–13)	0.922	11 (10–12)	10 (10–13)	11 (10–12)	0.843	
Absolute lymphocyte counts (cells /μL)	2535 (1575–4455)	2256(1617–3920)	2640(1523.5–4870.1)	0.402	2886 (1409.6–5017.5)	2525.2 (1725.3–5215.4)	2886 (1386–5030)	0.888	
Absolute neutrophil counts (cells /μL)	3553 (2150.5–6022)	3025 (1643.5–5641.5)	3624 (2262–6191)	0.121	3221 (1920–6900)	3715.5 (1610.3–8201.8)	3219 (1929–6831)	0.755	
CD3 + T cells (% of lymphocytes)	69.1 (51–84)	69 (58–81)	69.5 (48–84)	0.617	83 (46–90.2)	73 (60.5–89)	83 (41.3–91)	0.994	
CD4 + T cells (% of T cells)	33 (19.2–45)	33.6 (25–42.5)	33 (18–45.75)	0.265	39 (17.5–51)	44 (26–52.5)	36.5 (16–51)	0.168	
CD8 + T cells (% of T cells)	28.3 (17–40)	27 (18.25–37.75)	28.3 (16.65–40)	0.765	30 (12–41)	22.4 (15.4–41)	30 (12–41)	0.746	
CD16 + 56 + NK cells (% of lymphocytes)	10 (4–20)	7 (2.7–14.9)	10.25 (5.68–29)	0.013*	11 (6–32.5)	7.9 (3.8–12.9)	12 (6–41)	0.170	
CD19 + B cells (% of lymphocytes)	5.6 (0.5–16)	7 (2–16.8)	4.15 (0.3–16)	0.040*	0.5 (0–2)	1.1 (0–9.6)	0.5 (0–2)	0.192	
IgG (mg/dL)	350 (95.3–760)	298.5 (97–583.75)	407.5 (98–851.5)	0.064	171.5 (50.5–424)	252 (97.5–343.8)	158.5 (40.8–514.3)	0.853	
IgA (mg/dL)	9 (2–51.3)	8 (2–29)	10 (2–60)	0.156	9 (0.9–27.5)	6 (0–19.3)	9 (1–30)	0.298	
IgM (mg/dL)	59 (16–159)	42 (18–153)	64 (15–164)	0.791	19 (5–56.5)	22.1 (5–40)	19 (5–60)	0.757	
IgE (IU/mL)	3 (1–14)	1 (0–9.05)	3.05 (1–21)	0.019*	4 (1–12.8)	1 (1–2441)	4 (1–11.3)	0.645	

Parameters; median, (IQR)	CSR defects (*n* = 140)				Terminal B cell defects (*n* = 115)				*P* value
	Total	With autoimmunity	Without autoimmunity	*P* value	Total	With autoimmunity	Without autoimmunity	*P* value	
WBC × 1000 (cell/μL)	7.1 (4.9–10.7)	5.8 (3.4–9.6)	7.5 (5–11.1)	0.097	8.3 (5.8–12)	6.9 (5.3–8.7)	9.3 (6.6–13.1)	0.009*	0.069
Hemoglobin (g/dL)	11.9 (10–13)	11 (9.8–12.3)	12 (10.7–13)	0.157	12 (10–13)	12 (10–13)	11.7 (9.9–13)	0.343	0.114
Absolute lymphocyte counts (cells /μL)	2280 (1457.5–4060)	2294 (1475–4644)	2252 (1421.3–3905.5)	0.929	2507.7 (1754–4368)	2232 (1665.6–3294)	2957.6 (1844–5366.3)	0.074	0.451
Absolute neutrophil counts (cells /μL)	3081 (1872–5030)	2542.5 (729.5–3806)	3441 (2025–5258)	0.014*	3922 (2720–6228)	3652 (2514.3–5733)	4334.5 (2787–6606.3)	0.140	0.024*
CD3 + T cells (% of lymphocytes)	63.5 (50.7–76)	66 (51.3–79)	62.7 (50.4–75.3)	0.454	67 (53–77.5)	69 (57–78.5)	63.2 (49.4–76.8)	0.128	< 0.001*
CD4 + T cells (% of T cells)	30.7 (20–41)	30.8 (26.5–41.5)	30.4 (18–41)	0.247	32 (21–40)	32 (23.5–38.4)	32 (19.8–42)	0.868	0.085
CD8 + T cells (% of T cells)	25 (17.75–36.18)	21.3 (16.3–28.8)	27.3 (19.3–37.3)	0.058	29.75 (20–41.95)	31.3 (19.8–42)	28.4 (20–41.8)	0.527	0.130
CD16 + 56 + NK cells (% of lymphocytes)	8.5 (3.25–18)	4 (1.5–17.6)	9 (4–19)	0.250	10 (3.3–16.05)	8 (2–14.8)	10.6 (5.4–21.2)	0.163	0.163
CD19 + B cells (% of lymphocytes)	13.1 (5.9–20)	14.8 (4.3–24.9)	13 (6.2–19.7)	0.620	10 (3.95–18)	7.5 (3.9–13.8)	13 (3.5–21.5)	0.104	< 0.001*
IgG (mg/dL)	420 (81.3–819.5)	114 (26–453)	508 (107–879.5)	0.005*	603.5 (273.8–1001.5)	389 (199–675)	720 (358.5–1088.5)	0.006*	< 0.001*
IgA (mg/dL)	7 (2–26.3)	7.5 (1–19)	7 (3–30.8)	0.424	43.4 (5–140)	10 (3–58)	59.5 (6.25–187.3)	0.011*	< 0.001*
IgM (mg/dL)	180 (72–380)	192 (35–850)	174.5 (76.3–324.5)	0.635	55 (18–110)	44 (17–110)	60.5 (19.3–110)	0.582	< 0.001*
IgE (IU/mL)	1 (1–5.3)	1 (0–4)	1 (1–7)	0.034*	8.75 (1–425)	3 (0–13)	114 (2–1707)	< 0.001*	< 0.001*

*Hb* hemoglobin, *Ig* immunoglobulin, *NK cell* natural killer cell, *WBC* white blood cell

^*^ and bold values indicate *P* < 0.05 and are considered significant

The absolute lymphocyte count was significantly lower in patients with mixed intrinsic and extrinsic gene defects, in comparison with the intrinsic gene defect group (2219 cells/μL vs. 3528 cells/μL, *P* < 0.001). Patients in the intrinsic gene group had a significantly lower frequency of CD19 + B cells and CD16 + 56 + NK cells compared to the mixed intrinsic and extrinsic genes group (*P* < 0.001). The CD3 + , CD4 + , and CD8 + T cells had a higher frequency in patients with intrinsic gene defects than in the other group (*P* < 0.001, Table S5).

According to the immunologic profile of patients with mutations in the four main genes, the absolute lymphocyte count was significantly lower in patients with ATM defieincy compared to the other three gene defects (*P* < 0.001). In patients with *ATM* mutation, the IgG level was significantly lower in patients with autoimmunity than in those without (*P* < 0.001). In contrast, patients with ATM defects who had autoimmunity had a higher level of IgM compared to those without autoimmunity (*P* = 0.040).

**Fig. 4:**
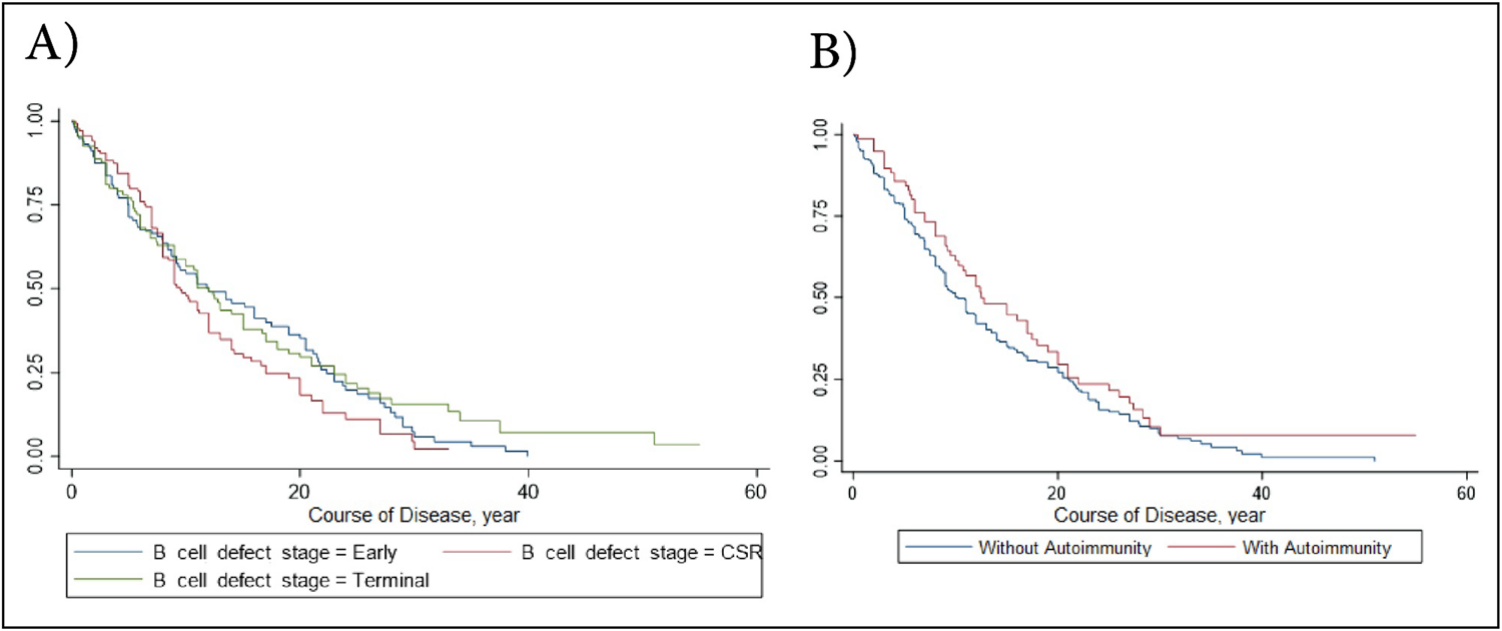
Kaplan–Meier survival analysis in patients with primary B cell defects. **A** Based on the B cell defect stage (early stage with 138 patients, CSR stage with 140 patients, and terminal stage patients with 115 patients). **B** Based on the autoimmunity presence (81 patients with autoimmunity and 312 without autoimmunity)

The Kaplan–Meier curves illustrated in Fig. 4 demonstrated that no significant differences were observed in the survival status of patients with B cell defects based on the B cell defect stage (*P* = 0.377) and autoimmunity presence (*P* = 0.10).

## Discussion

Autoimmune diseases may affect all subgroups of IEI, as reported with considerable frequency in patients with B cell defects [20, 21]. While autoimmunity is a well-known component of immune deficiency, there is an insufficient number of comprehensive studies on the prevalence of autoimmunity based on B cell development and differential stages. In the current study, we retrospectively investigated autoimmune manifestation in patients with intrinsic and extrinsic genetic defects in different stages of B cell development and compared the clinical, immunologic, and molecular characteristics between patients with and without autoimmunity in each stage. Our current findings on cases with intrinsic B cell defects showed that they have higher age and longer follow-up as well as lower mortality rate compared to patients with mixed/extrinsic defects. Although respiratory manifestations (infections and consequential bronchiectasis) and antibody production impairment/low B cell counts are more prominent in intrinsic B cell defects, severe/opportunistic infections (expect meningitis), other systemic complications, lymphoproliferation, and cellular immunity abnormality are frequently observed cases with mixed/extrinsic defects. Since the molecular pathogenesis of these groups is distinct, majority of previous research studied them separately [19, 22]; however, there are very little known about the comparison of B cell function and associated clinical manifestations between these two groups [23–25]. It has been reported that most of the B cell defects are caused by intrinsic causes, but some patients are secondary to functional impairments of other immune cell lineages (non-B cell–specific defect) [26]. This group includes impairments of T cell differentiation or the defect in T cell co-stimulatory molecules or abnormality in the generation, maintenance, or activation of T follicular helper cells (TFH). As more recently described, the phenomenon can also be caused by functional impairments in innate immune cells as defects in neutrophils or in Toll-like receptor (TLR) pathways [7, 19, 27].

The frequency of autoimmune manifestations varied in different IEI studies. In this cohort study, 20% of patients with monogenic B cell defects had a history of autoimmunity. In a study by Fischer et al., autoimmunity/inflammation was observed in 26.2% of patients with IEI [28], while in another study by Kaplan et al., autoimmune/inflammatory manifestations were observed in 10.1% of IEI cases [29]. The age at the onset and diagnosis of B cell defects was significantly higher in patients with autoimmunity than in others. Similarly, a recent study reported a considerably longer diagnostic delay in IEI patients with autoimmunity, which was related to the less severe non-infectious presentations in these patients [30]. These findings partly explain that the presence of autoimmunity might affect the diagnosis of the patients. The age at the onset of B cell defect in patients with the early-stage impairment was significantly lower compared to other stages, and delay in diagnosis was higher in patients with a defect in the terminal stage. We speculate that this might be due to the severity of manifestations and the higher rate of infection as the first presentation in the early stage and the higher rate of autoimmunity in the terminal stage, considering patients with autoimmunity had higher age of diagnosis.

Our patients with a defect in the terminal stage of B cell development had a higher frequency of autoimmunity (33.9% compared to 17.1% and 13% related to CSR and early stage, respectively). To our knowledge, no distinct study has investigated the prevalence of autoimmunity in each stage of B cell differentiation. However, various studies have been conducted to study the association between autoimmunity and a specific mutation responsible for genetic defects at each stage. Various literature reported that CVID was the most common primary antibody deficiency that occurred with autoimmunity, with an approximate ratio of 20–30% [16, 20, 31]. In former reports of CVID patients [32, 33], organ-specific autoimmune disease was diagnosed in 28.6% and 36.6% of subjects, respectively, with ITP as the most frequent autoimmunity in both publications. Autoimmunity phenotype is much less common in XLA than in other types of IEI [34]. According to Azizi et al., the autoimmunity rate in agammaglobulinemia was 12.7% [15]. In a survey, 69% of XLA patients reported at least one, and 53% reported multiple inflammatory symptoms. However, only 28% of patients were formally diagnosed with an inflammatory disease [35]. Among the patients with autoimmunity, 67.9% had only one type of autoimmunity, whereas 32.1% had poly-autoimmunity, most of whom had terminal stage defects. The most prevalent organ-specific autoimmunity was hematological, including ITP and AIHA. These findings are consistent with our prior studies, which found that the most common autoimmune manifestation among patients with IEI is autoimmune cytopenias [15, 36].

Among patients with a terminal stage mutation, ITP was the most common autoimmunity, while RA/JIA was the most prevalent autoimmunity in patients with a gene defect in the early stage. Various studies have reported that ITP is the most frequent manifestation in CVID patients [33, 37–39]. A review by Chawla et al. reported that the proportion of patients with CVID who develop ITP ranges from 7.4 to 19% and summarized the findings of various studies about immunopathogenesis associated with autoimmune cytopenia in patients with CVID. Some review studies found an increased proportion of CD21^low^ B cells and CD4 + HLA-DR + T cells in patients with CVID-associated autoimmune cytopenia [40]. In a cohort of 62 antibody-deficient patients, the expansion of CD21^low^ B cells was seen in antibody-deficient patients with non-infectious complications when presented as the frequency of total B cells but not in absolute cell numbers [41]. The current study showed a significant correlation between the increased frequencies of CD19 + B cells and autoimmunity. Previous studies demonstrated that rheumatologic involvement is the most frequent manifestation in patients with agammaglobulinemia and investigated the importance of BTK for human B cell tolerance and the role of its deficiency in systemic autoimmune diseases such as RA [42, 43]. Endocrine autoimmunity including insulin-dependent diabetes mellitus (IDDM) and autoimmune thyroiditis (AIT) was the least common manifestation (only five patients), and 80% of them were at the terminal stages. A previous cohort study investigated the role of the *LRBA* gene, involved in the terminal stage of B cell defects, in the etiology of neonatal diabetes [44].

It is noteworthy that autoimmune diseases could be the first or only clinical manifestations of the IEI diagnosis. In the current study, 3.8% of all patients showed an autoimmune complication as the first presentation of their IEI, 53.3% of whom were with the defects of terminal stage of B cells. As the first presentation, infection was reported in higher frequency in patients with a defect in the early stage. This study showed that 55.1% of IEI patients with autoimmune complications had the first episode of autoimmunity before the IEI diagnosis; in 34.6% of patients, IEI was diagnosed prior to autoimmunity, and in 10.3% of patients, IEI and autoimmunity were diagnosed at the same time. Our previous study and others have also reported that autoimmune manifestations are diagnosed before IEI in most patients with IEI [15, 29]. While in Massaad et al.’s study, 47% of autoimmune manifestations were among the presenting symptoms at the time of IEI diagnosis, 53% were documented after establishing the diagnosis [30]. Therefore, autoimmunity may be a warning sign of IEI, especially hematologic, and these patients could benefit from regular follow-up.

Mutations in various genes involved in B cell development and tolerance lead to impaired antibody production; thus challenging the detection of diagnostic auto-antibodies and documentation of the autoimmunity diagnosis [45]. In about 60% of our cases, immunodeficiency was caused by a mutation in 3 out of 37 identified genes, including *ATM*, *BTK*, and *LRBA*. Autoimmune diseases were more frequently present in patients with a mutation in the *LRBA* gene (> 70% of cases). More than half of LRBA-deficient patients with autoimmunity had poly-autoimmunity. In a previous study by Azizi et al., autoimmunity presented as poly-autoimmunity in 64.2% of patients, and autoimmune cytopenias were the most prevalent complication [46]. The spectrum of symptoms related to *LRBA* deficiency is vast and variable. In a cohort of 22 *LRBA*-deficient patients, the leading clinical complication of *LRBA* deficiency was autoimmune diseases (95%), particularly enteropathy, autoimmune hemolytic anemia, and ITP [47]. Regarding the biological role of *LRBA* protein in the immune system, it is thought to regulate the CTLA4 protein, an inhibitory immunoreceptor with a critical function in maintaining self-tolerance and regulatory T cells [48].

In conclusion, defects in different stages of B cell development can lead to different types of B cell defects with various autoimmune manifestations. Although further investigations are needed, this study contributes to a better understanding of the impact of mutation in the genes involved in different stages of B cell development in causing different types of autoimmune diseases. Our data suggest that the terminal stage and gene mutations involved in the terminal stage have the most association with autoimmunity compared to the other two stages.

## Supplementary Information

Below is the link to the electronic supplementary material.

Supplementary file1 (DOCX 84 KB)

## Data Availability

The datasets generated during and/or analyzed during the current study are available from the corresponding author on reasonable request.

## Abbreviations

AIHA: Autoimmune hemolytic anemia
AT: Ataxia-telangiectasia
ATM: Ataxia telangiectasia mutated
BTK: Bruton tyrosine kinase
CID: Combined immunodeficiency
CSR: Class switch recombination
CVID: Common variable immunodeficiency
HIES: Hyper-IgE syndrome
HIgM: Hyper-IgM syndrome
IBD: Inflammatory bowel diseases
IEI: Inborn errors of immunity
ITP: Immune thrombocytopenic purpura
JIA: Juvenile idiopathic arthritis
LRBA: Lipopolysaccharide-responsive and beige-like anchor
RA: Rheumatoid arthritis
SIgAD: Selective immunoglobulin A deficiency
TACI: Transmembrane activator and CAML interactor
SCID: Severe combined immunodeficiency
