# Supplementary material for: The Autoimmune Manifestations in Patients with Genetic Defects in the B Cell Development and Differentiation Stages
Source: J Clin Immunol. 2023 Feb 15;43(4):819–34. doi: 10.1007/s10875-023-01442-6 (PMC10110688; doi:10.1007/s10875-023-01442-6)
Supplement: Supplementary file 1 — Supplementary file1 (DOCX 84 KB) [file 10875_2023_1442_MOESM1_ESM.docx]

284 patients with mixed intrinsic and extrinsic B cell defects

109 patients with intrinsic B cell defects

- Early stage (n= 94)
- CSR stage (n= 10)
- Terminal stage (n= 5)

459 patients with known genetic defects in B cell registered in the national IEI registry

66 patients were excluded.

- missing data
- more than one gene defect

**Figure S1.** The inclusion/exclusion diagram of patients.

**Table S1.** The classification of intrinsic and mixed intrinsic and extrinsic genes.

| **Intrinsic Genes** | **Mixed Intrinsic and Extrinsic Genes** |
| --- | --- |
| *CD79A, CD79B, BLNK, IGHM, BTK, IGLL1, TCF3 (monoallelic GOF, biallelic LOF), TOP2B, LRRC8, FNIP1, AICDA, UNG, INO80, MSH2, MSH6, CTNNBL1, TNFRSF13C (encoded for TACI), TNFRSF13B (encoded for BAFFR), TNFSF12 (encoded for TWEAK), TNFSF13 (encoded for APRIL), CD19, CD81, MS4A1 (encoded for CD20), CR2 (encoded for CD21), IRF2BP2, SEC61A1, ATP6AP1, ARHGEF1, SH3KBP1, MOGS, IGKC.* | *PIK3R1, PIK3CD, IKZF1(encoded for IKAROS), RAG1, RAG2, DCLRE1C (encoded for ARTEMIS), PRKDC, NHEJ1, LIG4, TCN2, SLC46A1, MTHFD1, ADA, PNP, AK2, FANCI−23, DKC1-13, SAMD9L, SAMD9, SRP72, TP53, CD40, CD40L, RELA, GINS1, PMS2, ATM, MRE11, NBS, BLM, POLE1, POLE2, ERCC6L2, DNMT3B, ZBTB24, CDCA7, HELLS, IKBKG, NFKBIA, IKBKB, LRBA, CTLA4, STAT1, PTEN, BLK, NFKB1, NFKB2, BACH2, IL21, IL21R, RAC2, VAV1, CD27, CD70, CARD11, ICOS, ICOSL, PIK3CG, NIK, REL, MSN, STK4, DOCK8, WAS, MYSM1, FOXN1, RNU4ATAC, EXTL3, TTC7A, TTC37, SKIV2L, SP110, EPG5, RBCK1, RNF31, CCBE1, FAT4, NFE2L2, STAT5B, KMT2D, KDM6A, KMT2A, CXCR4 GOF, TPP2, SH2D1A, XIAP, CTPS1, RASGRP1, CARMIL2, MAGT1, PRKCD, PLCG2.* |

**Table S2.** Genetic variants detected in the cohort of 393 patients with primary B cell defects.

| **Gene symbol** | **Nucleotide change (Protein change)** | **Mutation type** | **Inheritance** (**Zigosity)** |
| --- | --- | --- | --- |
| *ATM* | **c. 8741T>A**(p.Ile2914Asn), **c.1159A>C**(p.Lys387Gln), **c.8741T>A**(p.Ile2914Asn), **c.5003T>G** (p.Leu1668Arg), **c.6047A>G** (p.Asp2016Gly), **c.6452G>C** (p.Arg2151Thr), **c.6744G>A** (p.Lys2248=), **c.7788G>A** (p.Glu2596=), **c.7865C>T** (p.Ala2622Val). | Point Mutation (Mis) | AR (Hom) |
|  | **c.829G>T**(p.Glu277Ter), **c.6658C>T**(p.Gln2220Ter), **c.664C>T** (p.Gln222Ter), **c.67C>T** (p.Arg23Ter), **c.4864G>T** (p.Glu1622Ter). | Point Mutation (Non) |  |
|  | **c.6199-1G>T**(-),**c.6807+1G>C**(-), **c.2921+1G>T**(-), **c.6453-2A>G**(-),**c.7308-6T>G**(-). | Point Mutation (Spl) |  |
|  | **c.3244_3245insG** (p.His1082ArgfsTer14), **c.5552_5553insC** (p.Gln1852ProfsTer5), **c.8375_8376insC** (p.Arg2792SerfsTer4). | Insertion |  |
|  | **c.3600_3601delTT** (p.Phe1201TrpfsTer3), **c.3895delG** (p.Ala1299ProfsTer50), **c.5585delT**(p.Ser1863LeufsTer54), **c.634delT** (p.Ser214ProfsTer16), **c.8046-8047delTA** (p.Ile2683ThrfsTer4), **c.7883delT** (p.Ile2629TyrfsTer2). **c.3602_3603delT**(p.Phe1201fs) | Deletion |  |
|  | **del EX61-EX62**, **del EX61-EX63**, **del EX61-EX64**, **del EX61-EX65**, **del EX62-EX63**, **del EX37-EX48**, **del EX59-EX60**, **del EX1**. | Large Deletion |  |
|  | **c.5712dupA** (p.Ser1905IlefsTer25), **c.9097_9101dupAATTT**(p.Leu3035IlefsTer8) | Duplication |  |
|  | **c.1537C>T** (p.Gln513Ter) and **c.8050C>T**(p.Gln2684Ter),  **c.6259delG**(p.Glu2087LysfsTer9) and **c.6658C>T** (p.Gln2220Ter),  **c.8907T>G**(p.Tyr2969Tyr) and **c.8050C>T**(p.Gln2684Ter),  **c.8907T>C**(p.Tyr2969Tyr) and **c.2639-1G>A**(-),  **dup EX18-EX61** and **c.7788G>A** (p.Glu2596=),  **c.7883delT** (p.Ile2629TyrfsTer2) and **c.7668delT** (p.Leu2557CysfsTer7),  **c.7883delT**(p.Ile2629TyrfsTer2) and **c.7655delA** (p.His2552ProfsTer12),  **c.8268G>C**(p.Lys2756Asn) and **c.7883delT**(p.Ile2629TyrfsTer2),  **c.8280delC**(p.Ser2761LeufsTer45) and **c.7883delT**(p.Ile2629TyrfsTer2). | 8 Deletion  4 Point Mutation  (Non)  3 Point Mutation (Mis)  1 Point Mutation (Spl)  1 Duplication  1 Silence mutation | AR (CH) |
| *BTK* | **c.1037T>C** (p.Leu346Pro), **c.777G>A** (p.Gly259Gly) **c.1651T>C** (p.Tyr551His), **c. 1424T>C** (p.Ile651Thr) **c.1048C>G** (p.His350Asp) **c.1631G>T** (p.Arg544Met), **c.1856C>T** (p.Pro619Leu), **c.1574G>A** (p.Arg525Gln), **c.1792T>A** (p.Tyr598Asn), **c.1239T>A** (p.Phe413Leu, **c.1697C>A** (p.Pro566Gln**), c.1978C>T** (p.Val626Leu) **c.83G>A** (p.Arg28His), **c.1526T>A** (p.Met509Lys), **c.214A>T** (p.Asn72Tyr)**, c.1427T>C** (p.Leu476Pro)**, c.1048C>G** (p.His350Asp), **c.1896T>A** (p.Ser632Arg), **c.895C>T** (p.Pro299Ser), **c.319G>T** (p.Asp107Tyr) | Point Mutation(Mis) | XLR (Hemi) |
|  | **c.1184G>A** (p.Trp395Ter) | Point Mutation (Non) |  |
|  | **c.141+5G>C**(-), **ivs17 + 5G>A**(-), **ivs14-1G>A**(-) | Point Mutation (Spl) |  |
|  | **c.349delA** (p.Thr117fs), **c.875delA** (p.Glu292fs), **c.1475del** (p.Arg492ProfsTer8) | Deletion |  |
| *LRBA* | **c.6607C>T** (p.Arg2203Cys)**, c.7742T>A** (p.M2581K) | Point Mutation (Mis) | AR (Hom) |
|  | **c.175G>T** (p.Glu59Ter)**, c.544C>T** (p.Arg182Ter)**, c.4814C>G** (p.Ser1605Ter)**, c.7009C>T** (p.Arg2337Ter) | Point Mutation (Non) |  |
|  | **c.1014+1G>A** (-)**, c4730-3T>G** (-), **c.4729+2dupT (-)** | Point Mutation (Spl) |  |
|  | **c.743_744insAAGA** (p.Asp248GlufsTer3) | Insertion |  |
|  | **c.2836_2839del** (p.Glu946Ter)**, c.5623del** (p.Ile1875SerfsTer14)**, c.2166_2766del** (p. V723SfsX25) | Deletion |  |
|  | **del EX41, del EX29-30, del EX1-2** | Large Deletion |  |
|  | **c.5581-5delT(-) and c.2920C>A** (p.Pro974Thr)  **c.5941C>T** (p.Arg1981Cys) **and c.3859A>G** (p.Ile1287Val) | 3 Point Mutation (Mis)  1 Deletion | AR (CH) |
| *CD40LG* | **c.499G>C** (p.Gly167Arg)**, c.482T>C** (p.Leu161Pro)**, c.464T>C** (p.Leu155Pro), **c.431G>T** (p.Gly144Val)**, c.655G>A** (p.Gly219Arg)**, c.107T>C** (p.Met36Thr)**, c.251G>C** (p.Cys84Ser) | Point Mutation (Mis) | XLR (Hemi) |
|  | **c.556C>T** (p.Gln186Ter)**, c.242T>A** (p.Leu81Ter) | Point Mutation (Non) |  |
|  | **c.156+2T>C (-)** | Point Mutation (Spl) |  |
|  | **c.266del** (p.Ser89ThrfsTer7)**, c.84del** (p.Thr29LeufsTer8)**, c.184_200del** (p.Asp62AsnfsTer18) | Deletion |  |
| *DOCK8* | **c.3577C>G** (p.Leu1193Val) | Point Mutation (Mis) | AR (Hom) |
|  | **c.4201G>T** (p.Glu1401Ter)**, c.1444C>T** (p.Arg482Ter)**, c.5132C>A** (p.Ser1711Ter) | Point Mutation (Non) |  |
|  | **c.4682+1G>T**(-)**, c.741+5G>T**(-)**, c.2555-1G>A**(-) | Point Mutation (Spl) |  |
|  | **p.R18fsX34,** | Insertion |  |
|  | **c.4106_4107insG** (p.Arg1370ProfsTer25) | Deletion |  |
|  | **Del EX9-12, del EX25-26, del EX1-10, del EX2-26** | Large Deletion |  |
|  | **c.3058A>G** (p.Ile1020Val) **and c.1623C>G** (p.His541Gln) | 2 Point Mutation (Mis) | AR (CH) |
| *RAG1* | **c.1073G>A** (p.Cys358Tyr)**, c.2570C>A** (p.Ala857Asp)**, c.2689C>T** (p.Arg897Ter), **c.1180C>T** (p.Arg394Trp)**, c.2564A>G** (p.Asn855Ser)**, c.2917C>T** (p.Arg973Cys)**, c.2521C>T** (p.Arg841Trp)**,** **c.1210C>T** (p.Arg404Trp) | Point Mutation (Mis) | AR (Hom) |
|  | **c.2985G>A** (p.Trp995Ter) | Point Mutation (Non) |  |
|  | **c.1405G>C** (p.Val469Leu) **and c.322C>T** (p.Arg108Ter) | 1 Point Mutation (Mis)  1 Point Mutation(Non) | AR (CH) |
| *DNMT3B* | **c.1823G>A** (p.Arg670Gln)**, c.1871A>G** (p.Glu624Gly)**, c.2428G>T** (Gly810Cys)**, c. 2356 G>A** (p.Glu806Lys)**, c.1878 T>A** (p.Ile646Ile) | Point Mutation(Mis) | AR (Hom) |
|  | **c.2397-11G>A** | Point Mutation (Spl) |  |
| *IGHM* | **c.1871A>G** (p.Tyr624Cys)**, c.1231T>G** (p.Tyr411Asp)**, c.56C>A** (p.Ser19Ter)**, c.206C>T** (p.Ser69Leu)**, c.734C>T** (p.Pro245Leu) | Point Mutation(Mis) | AR (Hom) |
|  | **c.358del** (p.Val120LeufsTer11) | Deletion |  |
|  | **c.68dup** (p.Asn24GlnfsTer60)**, c.525dup** (p.Tyr176LeufsTer78)**, c.188dup** (p.Lys64GlnfsTer104) | Duplication |  |
| *WAS* | **c.397G>A** (p.Glu133Lys)**, c.911G>C** (p.Arg304Pro)**, c.130T>A** (p.Leu44Met)**, c.257G>A** (p.Arg86His)**, c.91G>A** (p.Glu31Lys)**, c.391G>A** (p.Glu131Lys) | Point Mutation (Mis) | XLR (Hemi) |
|  | **c.961C>T** (p.Arg321Ter) | Point Mutation(Non) |  |
|  | **c.777+1G>A**(-)**, c.1453 +1G>C**(-) | Point Mutation (Spl) |  |
|  | **c.687del** (p.Lys230ArgfsTer31) | Deletion |  |
| *RAG2* | **c.685C>T** (p.Arg229Trp)**, c.130G>A** (p.Gly44Arg) | Point Mutation(Mis) | AR (Hom) |
| *ADA* | **c.704G>A** (p.Arg235Gln)**, c.845G>A** (p.Arg282Gln)**, c.445C>T** (p.Arg149Trp), **c.556G>A** (p.Glu186Lys)**, c.529G>A** (p.Val177Met)**, c.541G>A** (p.Asp181Asn) | Point Mutation(Mis) | AR (Hom) |
|  | **c.415G>T** (p.Glu139Ter)**, c.736C>T** (p.Gln246Ter) | Point Mutation(Non) |  |
|  | **c.778G>T** (p.Glu260Ter) **and c.821C>T** (p.Pro274Leu) | 1 Point Mutation (Mis)  1 Point Mutation(Non) | AR (CH) |
| *AICDA* | **c.334C>T** (p.Arg112Cys) | Point Mutation(Mis) | AR (Hom) |
|  | **c.364G>T** (p.Glu122Ter) | Point Mutation(Non) |  |
| *DCLRE1C* | **c.329T>G** (p.Leu110Arg)**, c.41G>T** (p.Gly14Val)**, c.632G>T** (p.Gly211Val) | Point Mutation(Mis) | AR (Hom) |
|  | **c.1162G>T** (p.Glu388Ter) | Point Mutation(Non) |  |
|  | **c.362+1G>T (-)** | Point Mutation (Spl) |  |
|  | **c.1250_1260del** (p.Ser417CysfsTer7) | Deletion |  |
|  | **del EX1-EX4** | Large Deletion |  |
| *ZBTB24* | **c.1224C>G** (p.Cys408Trp), **c.G1148C** (p.Cys383Ser), | Point Mutation (Mis) | AR (Hom) |
|  | **c.795dupA**(p.Asp266ArgfsTer28) | Deletion |  |
| *BAFFR* | **c.191G>T** (p.Gly64Val)**, c.475C>T** (p.His159Tyr) | Deletion | AR (Hom) |
| *CD27* | **c.287G>A** (p.Cys96Tyr)**, c.232C>T** (p.Arg78Trp)**, c.94T>C** (p.Tyr32His) | Point Mutation(Mis) | AR (Hom) |
| *PIK3R1* | **c.1020-8C>G(-), c.1425+1G>A(-)** | Point Mutation (Int) | AD (Het) |
| *RAC2* | **c. 167G>A** (p.Trp56Ter) | Point Mutation(Mis) | AR (Hom) |
| *XIAP* | **c.553G>A** (p.Ala185Thr)**, c.1408A>T** (p.Thr470Ser) | Point Mutation(Mis) | XLR (Hemi) |
|  | **c.664C>T** (p.Arg222Ter) | Point Mutation(Non) |  |
| *BLNK* | **c.679C>T** (p.Arg227Ter) | Point Mutation(Non) | AR (Hom) |
| *CD70* | **c.250del** (p.Ser84ProfsTer27) | Deletion | AR (Hom) |
| *CD79A* | **c.157C>T** (p.Gln53Ter) | Point Mutation(Non) | AR (Hom) |
|  | **c.233_234del** (p.Pro78ArgfsTer103) | Deletion |  |
| *CTLA4* | **c.436G>A** (p.Gly146Arg)**, c.326G>A** (p.Gly109Glu) | Point Mutation(Mis) | AD (Het) |
|  | **c.82delC** (p.Leu28PhefsTer44) | Deletion |  |
| *IKBKB* | **c.1106A>G** (p.Gln369Arg) | Point Mutation(Mis) | AR (Hom) |
|  | **c.2038C>T** (p.Arg680Ter) | Point Mutation(Non) |  |
| *NFKB2* | **c.1426G>A** (p.Gly476Arg) | Point Mutation(Mis) | AD (Het) |
|  | **c.459del** (p.Lys153AsnfsTer29) | Deletion |  |
| *NHEJ1* | **c.526C>T** (p.Arg176Ter)**, c.325C>T** (p.Arg109Ter) | Point Mutation(Non) | AR (Hom) |
| *PIK3CD* | **c.3061G>A** (p.Glu1021Lys)**, c.1558T>C** (p.Ser520Pro) | Point Mutation(Mis) | AR (Hom) |
| *STAT1* | **c.1688A>T** (p.Glu563Val)**, c.1154C>T** (p.Thr385Met)**, c.1232A>G** (p.Glu411Gly) | Point Mutation(Mis) | AR (Hom) |
| *CARD11* | **c.1009C>T** (p.Arg337Ter) | Point Mutation(Non) | AR (Hom) |
| *ICOS* | **c.451G>C** (p.Val151Leu) | Point Mutation(Mis) | AR (Hom) |
| *IKBKG* | **c.932A>G** (p.Asp311Gly) | Point Mutation(Mis) | XLR (Hemi) |
| *NFKB1* | **c.1736G>A** (p.Arg579Lys) | Point Mutation(Mis) | AD (Het) |
| *PRKCD* | **c.1293_1294insA** (p.Gly432ArgfsTer15) | Insertion | AR (Hom) |
| *SH2DA1* | **Ivs1+1G>A (-)** | Point Mutation (Spl) | XLR (Hemi) |
| *TTC7A* | **c.164C>T** (p.Ala55Val) | Point Mutation(Mis) | AR (Hom) |
| *TNFRSF13B* | **c.310T>C** (p.Cys104Arg) | Point Mutation(Mis) | AD (Het) |
| *TPP2* | **c.1270A>G** (p.Ile424Val) | Point Mutation(Mis) | AR (Hom) |

AD: Autosomal Dominant, AR: Autosomal Recessive, ARPC1B: Actin Related Protein 2/3 Complex Subunit 1B, ATM: ATM; Ataxia telangiectasia mutated, CARD11: Caspase Recruitment Domain family member 11, CH; Compound Heterozygous, DNMT3B: DNA Methyltransferase 3 Beta, Hemi: Hemizygous, Het: Heterozygous, Hom: Homozygous, IKBKG: Inhibitor of Nuclear Factor Kappa B kinase Regulatory Subunit Gamma, Int: Intronic, KMT2D: lysine Methyltransferase 2D, Mis: Missense, Non: Nonsense, PNP: Purine Nucleoside Phosphorylase, STAT3: Signal Transducer and Activator of Transcription 3. TTC7A: tetratricopeptide repeat domain 7A, WAS: WASP actin nucleation promoting factor, XLR: X-linked Recessive, ZBTB24: zinc finger and BTB domain containing 24, Mis: missense mutations, Non: Nonsense mutations, Spl: Splicing mutations.

**Table S3.** Demographic data in intrinsic and mixed gene groups.

| parameters | Mixed intrinsic and extrinsic (n=284) | Intrinsic  (n=109) | P -value |
| --- | --- | --- | --- |
| Sex ratio, M/F (n = 393) | 164/120 | 94/15 | **<0.001*** |
| Age, y, median (IQR)(n=387) | 11 (6-17) | 16 (7-24) | **<0.001*** |
| Age at onset, y, median  (IQR)(n = 384) | 1 (0.42-2) | 0.92 (0.33-2) | 0.489 |
| Age at diagnosis of  PID, y, median (IQR)(n=376) | 4 (1-7) | 4 (1-8.5) | 0.744 |
| Delay in diagnosis,  y, median (IQR)(n=374) | 1.75 (0.25-5) | 2.5 (0.42-5.08) | 0.236 |
| Course of disease,  y, median (IQR)(n=382) | 8.08 (3-14) | 9.5 (4.46-21.79) | **0.016*** |
| Consanguinity (%) (n = 384) | 218 | 47 | **<0.001*** |
| Dead/alive ratio**(n=381) | 74/198 | 9/99 | **<0.001*** |

* Bold values indicate P <0 .05 and are considered significant.

**Table S4.** Clinical manifestations in intrinsic and mixed gene groups.

| Parameters | Mixed intrinsic and extrinsic (n=284) | Intrinsic  (n=109) | p-value |
| --- | --- | --- | --- |
| Infectious manifestation, n (%) | 235 (84.2%) | 88 (81.5%) | 0.543 |
| Otitis media, n (%) | 94 (33.8%) | 55 (50.9%) | **0.002*** |
| Sinusitis, n (%) | 67 (24.1%) | 47 (43.1%) | **<0.001*** |
| Pneumonia, n (%) | 61 (56%) | 150 (53.8%) | 0.734 |
| Skin infection, n (%) | 62 (22.2%) | 4 (3.7%) | **<0.001*** |
| Candidiasis, n (%) | 43 (15.5%) | 2 (1.8%) | **<0.001*** |
| Conjunctivitis, n (%) | 24 (8.6%) | 20 (18.3%) | **0.012*** |
| Meningitis, n (%) | 16 (5.8%) | 19 (17.4%) | **0.001*** |
| Septicemia, n (%) | 7 (2.5%) | 0 | 0.198 |
| Septic arthritis, n (%) | 15 (5.4%) | 3 (2.8%) | 0.420 |
| Bronchiectasis, n (%) | 36 (12.9%) | 28 (25.7%) | **0.004*** |
| Neutropenia, n (%) | 28 (10.2%) | 8 (7.3%) | 0.443 |
| Failure to thrive, n (%) | 63 (22.7%) | 11 (10.1%) | **0.004*** |
| Splenomegaly, n (%) | 58 (20.9%) | 13 (11.9%) | **0.042*** |
| Hepatomegaly, n (%) | 46 (16.5%) | 13 (11.9%) | 0.276 |
| Lymphadenopathy, n (%) | 40 (14.4%) | 17 (15.6%) | 0.752 |
| Clubbing, n (%) | 20 (7.2%) | 15 (13.8%) | 0.050 |
| Malignancy, n (%) | 10 (3.6%) | 1 (0.9%) | 0.192 |
| Enteropathy, n (%) | 65 (23.4%) | 22 (20.3%) | 0.588 |
| Allergy/asthma, n (%) | 41 (14.7%) | 10 (9.2%) | 0.181 |

* Bold values indicate P <0 .05 and are considered significant.

**Table S5.** Immunologic profile in intrinsic and mixed gene groups.

| Parameters; median, (IQR) | Mixed intrinsic and extrinsic (n=284) | Intrinsic  (n=109) | p-value |
| --- | --- | --- | --- |
|  |  |  |  |
| WBC × 1000 (cell/μL) | 7.4 (5.2-11) | 9.7 (7.1-13.5) | **<0.001*** |
| Hemoglobin (g/dL) | 11 (10-13) | 12 (10-13) | 0.388 |
| Absolute lymphocyte counts  (cells /μL), | 2219 (1464-4060) | 3528 (2093-5134) | **<0.001*** |
| Absolute neutrophil counts  (cells /μL) | 3356.5 (2188-5572.5) | 4121 (2050-7125) | **0.106*** |
| CD3+ T cells (% of  lymphocytes) | 62 (45-75) | 87 (74-92) | **<0.001*** |
| CD4+ T cells (% of T cells) | 30 (16-39.3) | 44 (30.6-54) | **<0.001*** |
| CD8+ T cells (% of T cells) | 25 (15.8-37) | 34.5 (23-42) | **<0.001*** |
| CD16+56+ NK cells (% of  lymphocytes) | 11 (4-29.5) | 6.8 (4-12) | **0.023*** |
| CD19+ B cells (% of  lymphocytes) | 9.6 (2-19) | 0.5 (0-2) | **<0.001*** |
| IgG (mg/dL) | 489 (151.5-888.3) | 132.5 (23.8-353.3) | **<0.001*** |
| IgA (mg/dL) | 10 (3-66) | 9 (0-23) | **0.002*** |
| IgM ( mg/dL) | 81 (30-192.8) | 19 (5-60) | **<0.001*** |
| IgE (IU/mL) | 3 (1-30) | 1 (1-9) | 0.188 |

*Note: Abbreviations: Hb, hemoglobin; Ig, immunoglobulin; NK cell, natural killer cell; WBC, white blood cell*

** bold values indicate P <0 .05 and are considered significant.*

**Table S6.** Demographic data in patients with a mutation in main genes.

| parameters | BTK (n=76) | | | | ATM (n=85) | | | | LRBA (n=34) | | | | DOCK8 (n=33) | | | | P -value |
| --- | --- | --- | --- | --- | --- | --- | --- | --- | --- | --- | --- | --- | --- | --- | --- | --- | --- |
|  | **Total** | **With auto-immunity** | **Without auto-immunity** | **P-value** | **Total** | **With auto-immunity** | **Without auto-immunity** | **P-value** | **Total** | **With auto-immunity** | **Without auto-immunity** | **P-value** | **Total** | **With auto-immunity** | **Without auto-immunity** | **P-value** |  |
| Sex ratio, M/F | 76/0 | 9/0 | 67/0 | 1.0 | 38/47 | 4/9 | 34/38 | 0.368 | 17/17 | 11/13 | 6/4 | 0.708 | 15/18 | 0/1 | 15/17 | 1.000 | **<0.001*** |
| Age, y, median (IQR) | 16 (7-24) | 14 (7-29) | 16 (6-24) | 0.535 | 12 (9-15.75) | 10 (8.5-16.5) | 12 (9-16) | 0.568 | 13.5 (7.75-22) | 14 (6.75-20) | 12 (7.75-25.75) | 0.705 | 12 (8-15.5) | 19 (19-19) | 12 (7.5-15) | 0.364 | 0.265 |
| Age at onset, y, median  (IQR) | 1 (0.42-2) | 0.83 (0.43-2) | 1 (0.42-2) | 0.647 | 1 (0.9-2) | 2 (1-3.25) | 1 (0.8-2) | 0.069 | 1.5 (0.5-3) | 2 (0.6-3) | 0.79 (0.25-2.25) | 0.118 | 1.5 (0.85-3) | 10 (10-10) | 1.5 (0.8-3) | 0.125 | 0.126 |
| Age at diagnosis of  PID, y, median (IQR) | 4.75 (2-8) | 5 (2-7) | 4.5 (2-8) | 0.960 | 5 (3-7) | 5 (3-7) | 5 (2.86-7.25) | 0.850 | 7 (3.75-12.25) | 7.5 (4-12.75) | 5 (1-11.5) | 0.287 | 6 (3-10) | 10 (10-10) | 6 (3-9.25) | 0.452 | 0.068 |
| Delay in diagnosis,  y, median (IQR) | 2.75 (0.75-5.46) | 3 (1.33-6.42) | 2.67 (0.63-5.06) | 0.534 | 3 (1-5) | 2 (0.25-4) | 3 (1-5) | 0.282 | 4.5 (1.5-7.5) | 4.75 (2.18-7.5) | 2 (0.7-9.5) | 0.677 | 4 (0.5-5.1) | 0.0 (0.0-0.0) | 4 (0.73-5.31) | 0.194 | 0.303 |
| Course of disease,  y, median (IQR) | 11(4.9-23) | 12 (6.5-27.87) | 11 (4.64-21.96) | 0.387 | 9.5 (7-14) | 9 (6.05-11) | 10 (7-15) | 0.186 | 11.2 (5.48-19.5) | 13.75 (5.6-20.5) | 7.15(0.44-19.25) | 0.287 | 10.5 (3.6-13) | 9 (9-9) | 11 (3.33-13) | 0.875 | 0.442 |
| Consanguinity (%) | 21 (27.6%) | 2 (22.7%) | 19 (28.4%) | 1.000 | 69 (83.1%) | 10 (76.9%) | 59 (84.3%) | 0.686 | 30 (90.9%) | 22 (91.7%) | 8 (88.9%) | 1.000 | 28 (87.5) | 0 | 28 (90.3%) | 0.635 | **<0.001*** |
| Dead/alive ratio** | 5/71 | 0/9 | 5/62 | 1.000 | 25/60 | 4/9 | 21/51 | 1.000 | 8/26 | 8/16 | 0/10 | 0.072 | 3/28 | 0/1 | 3/27 | 1.000 | **0.001*** |

*****bold values indicate P <0 .05 and are considered significant.

**Table S7.** Autoimmune manifestation in patients with a mutation in main genes.

| Parameters | BTK (n=76) | | | | ATM (n=85) | | | | LRBA (n=34) | | | | DOCK8 (n=33) | | | | p-value |
| --- | --- | --- | --- | --- | --- | --- | --- | --- | --- | --- | --- | --- | --- | --- | --- | --- | --- |
|  | **Total** | **Mono-autoimmunity** | **Poly-autoimmunity** | **P-value** | **Total** | **Mono-autoimmunity** | **Poly-autoimmunity** | **P-value** | **Total** | **Mono-autoimmunity** | **Poly-autoimmunity** | **P-value** | **Total** | **Mono-autoimmunity** | **Poly-autoimmunity** | **P-value** |  |
| Autoimmunity | 9 (11.8%) | 7 | 2 | **0.013*** | 13 (15.3%) | 10 | 3 | **0.003*** | 24 (70.6%) | 11 | 13 | **0.005*** | 1 (3%) | 1 | 0 | .... | **<0.001*** |
| Immune thrombocytopenic purpura | 1 (1.3%) | 0 | 1 | **0.026*** | 6 (7.1%) | 4 | 2 | **0.012*** | 13 (38.2%) | 3 | 10 | **0.001*** | 0 (0%) | 0 | 0 | .... | **<0.001*** |
| Autoimmune hemolytic anemia | 0 (0%) | 0 | 0 | .... | 4 (4.7) | 2 | 2 | **0.005*** | 8 (23.5%) | 0 | 8 | **<0.001*** | 0 (0%) | 0 | 0 | .... | **<0.001*** |
| Autoimmune enteropathy | 0 (0%) | 0 | 0 | .... | 0 (0%) | 0 | 0 | .... | 5 (14.7%) | 1 | 4 | 0.059 | 0 (0%) | 0 | 0 | .... | **<0.001*** |
| Rheumatoid arthritis/juvenile idiopathic arthritis | 7 (9.2%) | 5 | 2 | **0.007*** | 2 (2.4%) | 0 | 2 | **0.001*** | 5 (14.7%) | 4 | 1 | 0.627 | 0 (0%) | 0 | 0 | .... | **0.020*** |
| Autoimmune thyroiditis | 0 (0%) | 0 | 0 | .... | 0 (0%) | 0 | 0 | .... | 2 (5.9%) | 0 | 2 | 0.139 | 0 (0%) | 0 | 0 | .... | **0.010*** |
| Vitiligo | 1 (1.3%) | 1 | 0 | 1.000 | 0 (0%) | 0 | 0 | .... | 2 (5.9%) | 0 | 2 | 0.139 | 1 (3%) | 1 | 0 | 1.000 | 0.153 |
| Insulin-depended  diabetes mellitus | 0 (0%) | 0 | 0 | .... | 0 (0%) | 0 | 0 | .... | 1 (2.9%) | 1 | 0 | 1.000 | 0 (0%) | 0 | 0 | .... | 0.125 |
| Celiac disease | 0 (0%) | 0 | 0 | .... | 0 (0%) | 0 | 0 | .... | 2 (5.9%) | 1 | 1 | 1.000 | 0 (0%) | 0 | 0 | .... | **0.009*** |
| Inflammatory bowel disease | 0 (0%) | 0 | 0 | .... | 3 (3.5%) | 2 | 1 | 0.103 | 4 (11.8%) | 1 | 3 | 0.274 | 0 (0%) | 0 | 0 | .... | **0.007*** |
| Myasthenia gravis | 0 (0%) | 0 | 0 | .... | 0 (0%) | 0 | 0 | .... | 1 (2.9%) | 0 | 1 | 0.382 | 0 (0%) | 0 | 0 | .... | 0.125 |
| Systemic lupus erythematous | 0 (0%) | 0 | 0 | .... | 1 (1.2%) | 0 | 1 | **0.035*** | 0 (0%) | 0 | 0 | .... | 0 (0%) | 0 | 0 | .... | 0.639 |
| Psoriasis | 0 (0%) | 0 | 0 | .... | 1 (1.2%) | 1 | 0 | 1.000 | 0 (0%) | 0 | 0 | .... | 0 (0%) | 0 | 0 | .... | 0.639 |
| Kawasaki disease | 2 (2.6%) | 1 | 1 | 0.052 | 0 (0%) | 0 | 0 | .... | 0 (0%) | 0 | 0 | .... | 0 (0%) | 0 | 0 | .... | 0.263 |
| Evans syndrome | 0 (0%) | 0 | 0 | .... | 0 (0%) | 0 | 0 | .... | 2 (5.9%) | 0 | 2 | 0.139 | 0 (0%) | 0 | 0 | .... | **0.009*** |
| Multiple sclerosis | 0 (0%) | 0 | 0 | .... | 0 (0%) | 0 | 0 | .... | 1 (2.9%) | 0 | 1 | 0.382 | 0 (0%) | 0 | 0 | .... | 0.125 |
| Autoimmune hepatitis | 0 (0%) | 0 | 0 | .... | 1 (1.2%) | 1 | 0 | 1.000 | 1 (2.9%) | 0 | 1 | 0.382 | 0 (0%) | 0 | 0 | .... | 0.437 |

Note: * bold values indicate P <0 .05 and are considered significant.

**Table S8.** Clinical manifestation in patients with a mutation in main genes.

| Parameters | BTK (n=76) | | | | ATM (n=85) | | | | LRBA (n=34) | | | | DOCK8 (n=33) | | | | p-value |
| --- | --- | --- | --- | --- | --- | --- | --- | --- | --- | --- | --- | --- | --- | --- | --- | --- | --- |
|  | **Total** | **With autoimmunity** | **Without autoimmunity** | **P-value** | **Total** | **With autoimmunity** | **Without autoimmunity** | **P-value** | **Total** | **With autoimmunity** | **Without autoimmunity** | **P-value** | **Total** | **With autoimmunity** | **Without autoimmunity** | **P-value** |  |
| Infectious manifestation, n (%) | 60 (80%) | 6 | 54 | 0.372 | 67 (80.7%) | 12 | 55 | 0.446 | 34 (100%) | 24 | 10 | .... | 26 (81.3%) | 0 | 26 | 0.188 | **0.047*** |
| Otitis media, n (%) | 40 (52.6%) | 4 | 36 | 0.726 | 32 (38.6%) | 7 | 25 | 0.233 | 15 (44.1%) | 12 | 3 | 0.451 | 12 (36.4%) | 0 | 12 | 1.000 | 0.263 |
| Sinusitis, n (%) | 35 (46.1%) | 6 | 29 | 0.287 | 14 (16.5%) | 3 | 11 | 0.686 | 16 (47.1%) | 12 | 4 | 0.715 | 8 (24.2%) | 0 | 8 | 1.000 | **<0.001*** |
| Pneumonia, n (%) | 43 (56.8%) | 4 | 39 | 0.490 | 35 (41.2%) | 6 | 29 | 0.768 | 23 (67.6%) | 18 | 5 | 0.232 | 19 (57.6%) | 0 | 19 | 0.387 | **0.045*** |
| Skin infection, n (%) | 2 (2.6%) | 0 | 2 | 1.000 | 19 (22.4%) | 4 | 15 | 0.482 | 2 (5.9%) | 2 | 0 | 1.000 | 18 (54.6%) | 0 | 18 | 0.419 | **<0.001*** |
| Candidiasis, n (%) | 1 (1.3%) | 0 | 1 | 1.000 | 4 (4.7%) | 1 | 3 | 0.501 | 9 (26.5%) | 8 | 1 | 0.225 | 9 (27.3%) | 0 | 9 | 1.000 | **<0.001*** |
| Conjunctivitis, n (%) | 14 (18.4%) | 1 | 13 | 1.000 | 8 (9.4%) | 1 | 7 | 1.000 | 4 (11.8%) | 4 | 0 | 0.296 | 3 (9.1%) | 0 | 3 | 1.000 | 0.367 |
| Meningitis, n (%) | 17 (22.4%) | 1 | 16 | 0.674 | 3 (3.5%) | 0 | 3 | 1.000 | 4 (11.8%) | 3 | 1 | 1.000 | 5 (15.2%) | 0 | 5 | 1.000 | **0.005*** |
| Septicemia, n (%) | 0 | 0 | 0 | .... | 2 (2.4%) | 1 | 1 | 0.290 | 2 (5.9%) | 2 | 0 | 1.000 | 0 | 0 | 0 | .... | 0.146 |
| Septic arthritis, n (%) | 1 (1.3%) | 0 | 1 | 1.000 | 4 (4.7%) | 1 | 3 | 0.501 | 5 (14.7%) | 5 | 0 | 0.291 | 2 (6.1%) | 0 | 2 | 1.000 | **0.038*** |
| Bronchiectasis, n (%) | 20 (26.3%) | 2 | 18 | 1.000 | 4 (4.7%) | 0 | 4 | 1.000 | 12 (35.3%) | 11 | 1 | 0.061 | 5 (15.2%) | 0 | 5 | 1.000 | **<0.001*** |
| Neutropenia, n (%) | 7 (9.2%) | 7 | 0 | 0.589 | 5 (5.9%) | 2 | 3 | 0.173 | 0 | 0 | 0 | 1.000 | 1 (3%) | 0 | 1 | 1.000 | 0.284 |
| Failure to thrive, n (%) | 4 (5.3%) | 1 | 3 | 0.403 | 12 (14.1%) | 2 | 10 | 1.000 | 10 (29.4%) | 9 | 1 | 0.215 | 5 (15.2%) | 0 | 5 | 1.000 | **0.008*** |
| Splenomegaly, n (%) | 7 (9.2%) | 1 | 6 | 1.000 | 10 (11.8%) | 4 | 6 | **0.046*** | 21 (61.8%) | 19 | 2 | **0.002*** | 2 (6.1%) | 0 | 2 | 1.000 | **<0.001*** |
| Hepatomegaly, n (%) | 7 (9.2%) | 0 | 7 | 0.589 | 8 (9.4%) | 4 | 4 | **0.019*** | 12 (35.3%) | 11 | 1 | 0.061 | 6 (18.2%) | 0 | 6 | 1.000 | **0.001*** |
| Lymphadenopathy, n (%) | 9 (11.8%) | 1 | 8 | 1.000 | 6 (7.1%) | 2 | 4 | 0.236 | 11 (32.4%) | 11 | 0 | **0.014*** | 1 (3%) | 0 | 1 | 1.000 | **0.001*** |
| Clubbing, n (%) | 8 (10.5%) | 0 | 8 | 0.585 | 3 (3.5%) | 0 | 3 | 1.000 | 7 (20.6%) | 6 | 1 | 0.644 | 0 | 0 | 0 | .... | **0.005*** |
| Malignancy, n (%) | 1 (1.3%) | 1 | 0 | 0.118 | 6 (7.1%) | 1 | 5 | 1.000 | 0 | 0 | 0 | .... | 1 (3%) | 0 | 1 | 1.000 | 0.132 |
| Enteropathy, n (%) | 18 (23.7%) | 4 | 14 | 0.203 | 4 (4.7%) | 1 | 3 | 0.501 | 21 (61.8%) | 20 | 1 | **<0.001*** | 5 (15.2%) | 0 | 5 | 1.000 | **<0.001*** |
| Allergy/asthma, n (%) | 6 (7.9%) | 1 | 5 | 0.544 | 7 (8.2%) | 1 | 6 | 1.000 | 9 (26.5%) | 8 | 1 | 0.225 | 13 (39.4%) | 0 | 13 | 1.000 | **<0.001*** |

Note: * bold values indicate P <0 .05 and are considered significant.

**Table S9.** Immunologic profile in patients with a mutation in main genes.

| Parameters; median, (IQR) | BTK (n=76) | | | | ATM (n=85) | | | | LRBA (n=34) | | | | DOCK8 (n=33) | | | | p-value |
| --- | --- | --- | --- | --- | --- | --- | --- | --- | --- | --- | --- | --- | --- | --- | --- | --- | --- |
|  | **Total** | **With autoimmunity** | **Without autoimmunity** | **P-value** | **Total** | **With autoimmunity** | **Without autoimmunity** | **P-value** | **Total** | **With autoimmunity** | **Without autoimmunity** | **P-value** | **Total** | **With autoimmunity** | **Without autoimmunity** | **P-value** |  |
| WBC × 1000 (cell/μL) | 9.63 (7.17-14.20) | 12 (9.24-16.10) | 9.4 (7.1-13) | 0.101 | 5.74 (4.59-8.64) | 6.54 (2.87-10.35) | 5.74 (4.63-8.28) | 0.989 | 8.01 (5.43-13.83) | 7.9 (5.2-14.6) | 8 (6.2-9.8) | 0.881 | 8.89 (6.83-12.3) | 8.1 | 9.1 (6.8-12.7) | 0.880 | **<0.001*** |
| Hemoglobin (g/dL) | 12 (10-13) | 10 (9.5-13) | 12 (10.77-12.9) | 0.377 | 12 (10-13) | 11 (10-12.75) | 12.3 (11-13) | 0.164 | 12 (10-13) | 12 (10-13) | 12 (9.5-13) | 0.979 | 11.1 (8.98-12.53) | .... | 11.1 (9-12.5) | .... | 0.218 |
| Absolute lymphocyte counts  (cells /μL), | 3604 (2080-5145) | 3209 (1560-5698) | 3704.6 (2150.5-5148.8) | 0.532 | 1802 (1059.61-2535) | 1973 (1303.47-4081) | 1802.2 (1008 -2496) | 0.439 | 2213 (1644.9-44233) | 2055 (1617.8-3517.5) | 3322.5 (2162.4-5480.3) | 0.170 | 2559.3 (1706.8-4146) | 4368 | 2494.6 (1653-3480) | 0.417 | **<0.001*** |
| Absolute neutrophil counts  (cells /μL) | 4315.5 (2065-7068) | 7510 (2213-9648.5) | 3566.5 (2065-6845) | 0.231 | 3127 (2179-5246) | 3408 (945-4536) | 3117.5 (2188-5264) | 0.504 | 3891.5 (2989-6023) | 4877 (2519.5-6458) | 3857 (3652-4416) | 0.678 | 4492.5(2620.8-6741.8) | .... | 4592.5 (2620.8-6741.8) | .... | 0.202 |
| CD3+ T cells (% of  lymphocytes) | 89 (81-92) | 79 (62.5-89) | 89 (83-92.9) | 0.067 | 56.9 (42.5-74) | 67.5 (45.13-82) | 56.7 (42.3-69.25) | 0.270 | 69.5 (57-78.5) | 69 (55.5-76.6) | 72 (58.4-81.6) | 0.623 | 56 (47-67.45) | 56 | 57.15 (46.5-67.7) | 0.966 | **<0.001*** |
| CD4+ T cells (% of T cells) | 44 (34-54) | 39.6 (21.75-48.13) | 46.1 (35-54) | 0.249 | 28 (18-38) | 33 (26.66-41.25) | 24 (17-35.8) | 0.148 | 31.5 (23.5-37.63) | 31 (21.5-39.1) | 33.5 (30-37.4) | 0.564 | 29.1 (14.02-38.25) | 36 | 28.1 (13.7-39) | 0.571 | **<0.001*** |
| CD8+ T cells (% of T cells) | 35 (24-44) | 35 (20.5-44.75) | 34 (24.5-44.5) | 0.739 | 23 (18-37) | 21 (13.5-26.5) | 25 (18.75-38.03) | 0.266 | 30.5 (19-45.5) | 30.5 (19-47.5) | 31.7 (18.9-43.5) | 0.881 | 26 (18-42) | 18 | 26.5 (19-42.1) | 0.519 | **0.028*** |
| CD16+56+ NK cells (% of  lymphocytes) | 6 (3-11) | 17.1 (4-) | 6 (3-9.75) | 0.215 | 8.5 (1.55-18.25) | 9.55 (1.25-17.76) | 8.5 (1.55-21) | 0.837 | 10 (3-18) | 9.5 (2.3-17.3) | 10 (7-…) | 0.634 | 11.95 (7.08-28.15) | .... | 12 (7.1-28.2) | .... | 0.211 |
| CD19+ B cells (% of  lymphocytes) | 0 (0.0-1.5) | 0.55 (0-20) | 0 (0-1) | 0.242 | 10 (4.4-16.2) | 8.4 (2.8-15.5) | 11.2 (4.75-16.85) | 0.534 | 8 (4-13) | 7.5 (3.3-12.5) | 11.2 (8-14) | 0.391 | 17 (9.25-26.5) | 7 | 18 (10.4-27) | 0.462 | **<0.001*** |
| IgG (mg/dL) | 125 (28-348) | 160 (20-298) | 122.5 (36.25-401.25) | 0.610 | 685 (266.5-955.5) | 98 (31.25-389.75) | 745 (484.5-974) | **<0.001*** | 351 (135.25-849) | 325 (111.8-583.8) | 908.5 (395-1183.8) | 0.041 | 1067 (673-1548) | 1400 | 1003.5 (634.5 -1554.8) | 0.645 | **<0.001*** |
| IgA (mg/dL) | 9 (0-26.75) | 7 (1-24.5) | 9 (0-27.5) | 0.797 | 7 (2-32.5) | 5.5 (1.25-15.75) | 7 (3-47.25) | 0.339 | 9 (3-59.25) | 8 (2.3-37.8) | 45.5 (4.5-125.3) | 0.188 | 190 (91-350) | 55 | 190 (96.3-362.5) | 0.387 | **<0.001*** |
| IgM ( mg/dL) | 19 (8-40) | 24 (10.5-34.5) | 18.5 (7.75-55.75) | 0.979 | 192 (99.5-331.5) | 480 (119.25-978.25) | 184 (99-274) | **0.040*** | 67 (18.25-147.25) | 47 (18.3-136.3) | 113.5 (23-151.8) | 0.404 | 61 (31-110) | 74 | 60.5 (30.8-115.8) | 0.774 | **<0.001*** |
| IgE (IU/mL) | 1 (0.9-9) | 1 (1-2485.3) | 2 (0.1-9) | 0.459 | 1 (1-4.8) | 1 (0.02-3.5) | 1 (1-5) | 0.368 | 1 (0-7) | 0.7 (0-4.8) | 3.5 (1-71.5) | 0.070 | 1550 (324-2808) | 2500 | 1500 (279-2872) | 0.667 | **<0.001*** |

Note: * bold values indicate P <0 .05 and are considered significant.
